# Development of response-controlled tall buildings through own-mass mobilisation

**DOI:** 10.1038/s41467-026-74868-2

**Published:** 2026-06-24

**Authors:** Miguel Martinez-Paneda, Ahmed Y. Elghazouli, Kevin Gouder, William Algaard

**Affiliations:** 1 Department of Civil & Environmental Engineering, Imperial College London, London, UK; 2 Arup, London, UK; 3 Department of Civil & Environmental Engineering, The Hong Kong Polytechnic University, Hong Kong, Hong Kong; 4 Nile University, Giza, Egypt; 5 Department of Aeronautics, Imperial College London, London, UK

**Keywords:** Mechanical engineering, Civil engineering

## Abstract

This paper describes a structural response reduction approach that, rather than adding mass as in conventional mass damping techniques, takes advantage of the large inherent mass present within a building and uses its own mass to generate large levels of reliable damping with minimal differential movements and without the need for frequency tuning. The proposed concept challenges the traditional premise of conceiving tall buildings as rigid, static entities, and exploits movements to improve performance. The underlying premise is investigated using purpose-devised aeroelastic wind tunnel tests on a dynamically scaled model of a 300-metre prototype building in which the damping system is explicitly incorporated. The results show reductions exceeding 70% in peak accelerations and 50% in base moments, relative to a conventional undamped configuration, while maintaining differential displacements between the movable floors and the core below 50 mm under the 50-year return wind. The results of the wind tunnel tests, coupled with complementary numerical studies as well seismic time-history simulations, demonstrate the considerable promise of the proposed system for significantly reducing the demands on the superstructure and foundation. This opens the door for low-carbon, high-performance tall building designs that can enable more resilient and sustainable urban development.

## Abstract (web summary)

Authors explore an alternative mass damping technique. The study demonstrates that mobilising a portion of a tall building’s own mass using spring-damper connections reduces wind and seismic responses, achieving over 70% reduction in peak accelerations and 50% in base moments, with minimal differential floor movements.

## Introduction

With the rise in global urban population, designing tall buildings that are safe, resilient, and resource-efficient has become central to creating sustainable urban infrastructure^1,2^, particularly in developing economies^3,4^ with a high risk from extreme wind in a changing climate, and/or seismic loads^5–8^. Tall building construction has grown exponentially in recent decades, a trend that is expected to continue^9^. Despite the significant upfront investment, the benefits of tall buildings in terms of efficient land use, holistically reduced carbon footprint, increased urban density, and local economic growth, remain compelling^10,11^.

Tall building design is typically governed by the dynamic response to wind and seismic excitations, and more specifically by the requirement to ensure that wind-induced accelerations remain within user comfort limits^12–15^. Contrary to traditional design methods that control accelerations by increasing the building mass and stiffness, it is now widely recognised that using supplementary damping systems results in more optimal and resilient solutions^16–19^. This recognition is additionally driven by the significant non-structural architectural damage, repair costs, and interruption observed after recent seismic events^20,21^. The severe damage associated with dynamic events^15,20–25^, including the recent collapse of the State Audit Office tower in Bangkok^26^, has also shifted the focus further towards enhanced safety and resilience^27,28^. Existing damping techniques, however, exhibit various shortcomings that constrain their benefits and limit their applicability. For example, mass systems are generally unable to reduce wind or seismic strength demands, while distributed systems require large interventions and have a height limit of applicability due to the reduced inter-storey drifts in tall buildings^10,16–19,29^.

Here, we present a response-reduction approach that departs from the traditional premise of conceiving tall buildings as relatively rigid, static entities and proposes the notion of accepting and embracing building movements as a design asset. This design shift challenges the slow and scarce innovation in the construction industry in the last century^30^, to enable efficient and resilient tall buildings that can safely and economically support global urban growth. The dynamic response characteristics of the proposed system are established from first principles, supported by numerical simulations under wind and seismic excitations. Experimental validations, explicitly incorporating the approach, are also carried out, with a focus on advanced aeroelastic wind tunnel tests.

### Large own-mass damping

The proposed response reduction concept exploits the large inherent own mass of the building, rather than adding mass, in order to generate significant levels of reliable damping with minimal differential movements. To introduce the approach, it is useful to start from the underlying principle of mass-based damping. Conventional Tuned Mass Dampers (TMDs)^31^, such as that used in the Taipei 101 tower^18^, are arguably the most common amongst existing damping techniques, due to their relative simplicity and adaptability for inclusion at a late design stage, depending on wind tunnel test results^16^. However, despite their benefits^18,31^, TMDs have several limitations, including their inability to control seismic demands due to frequency tuning, as well as their shortcomings in reducing strength demands due to their lack of redundancy, as the system could be under maintenance in addition to possible detuning risks at the vibration levels associated with ultimate strength demands. The need to add a large extra mass, typically at the top of the building, also results in significant area and value loss, construction disruption and bespoke strengthening.

**Fig. 1:**
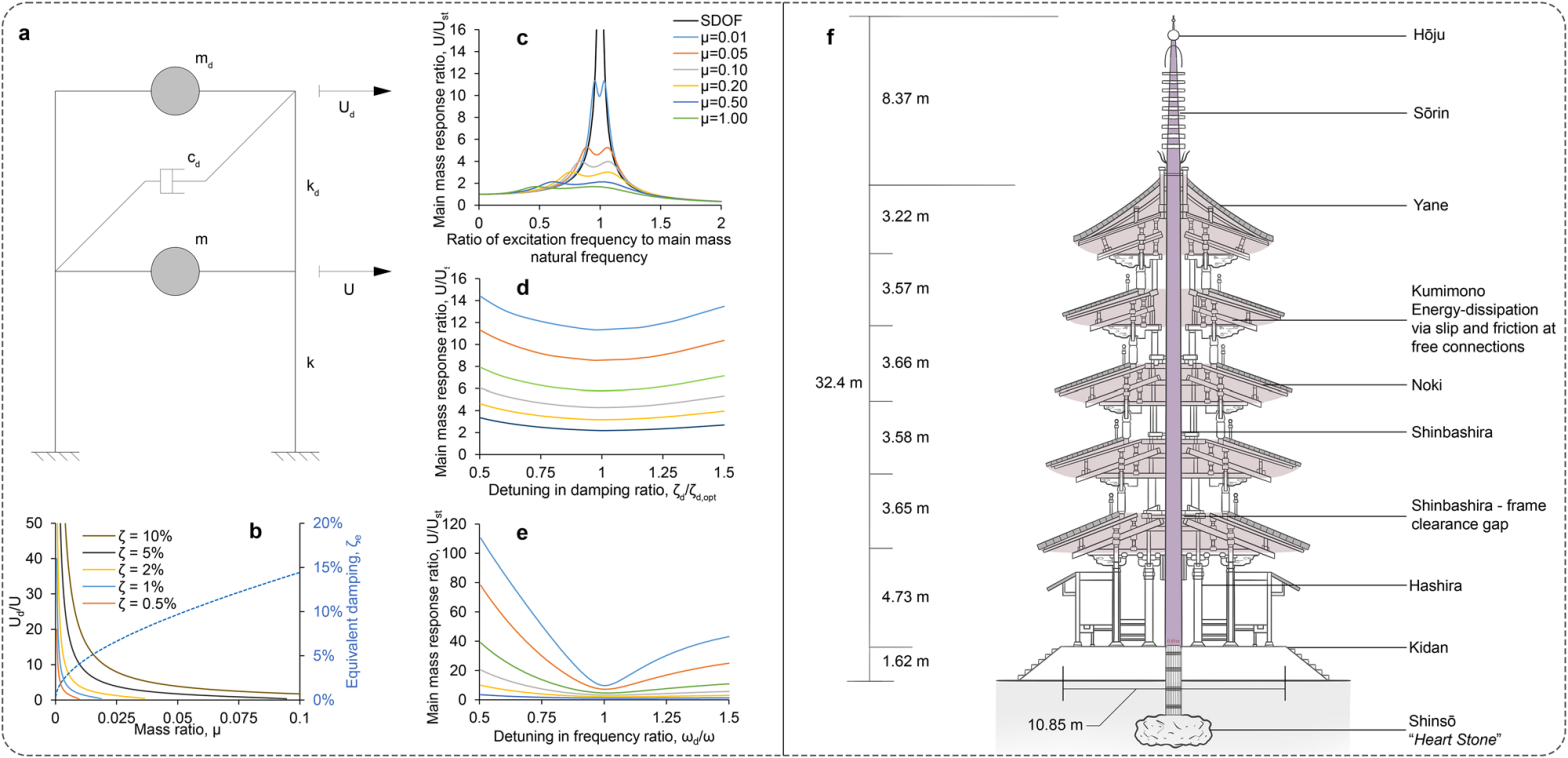
Large-mass damper behaviour. **a** Simplified 2DOF system of an SDOF with an attached TMD, where m is the mass, k the stiffness, c the damping, and U the displacement of the main mass, and subscript _d_ refers to damped mass parameters. **b** Variation in the ratio of maximum displacement amplitudes vs target equivalent damping, and equivalent damping under harmonic vibration for increasing mass ratio. **c** Dimensionless response of the system with different mass ratios under harmonic main mass excitation. **d** Detuning in damper mass damping ratio; and **e** detuning in damper mass frequency ratio. **f** Cross-section of the Horyu-ji Five-Storey Pagoda in Nara, Japan.

Although the ratio between the mass of the damper (i.e., mobilised mass) and that of the building is the parameter most influencing the TMD performance, it cannot practically exceed 1–2% of the large inherent mass of the structure. The crucial influence of this mass ratio on the response can be examined using the idealisation of a structure as a single-degree-of-freedom (SDOF), with an attached TMD (Fig. 1a). As shown in Fig. 1b, the equivalent damping that can be generated is directly related to the extent of mass mobilisation. As the damper mass increases, for a target damping level, the differential displacement between both masses reduces^29,32^. The benefits of mobilising a large damper mass have been explored in several seismic-oriented TMD and multiple tuned-mass damper (MTMD) variations, although these are hindered by constructability and integration constraints including large relative displacement and tuning requirements^18,19,33–45^. Mahmoud and Chlawat investigate the option of using a series of “suspended slab systems” to, doubling up the floor slabs, and reduce overall building response by combining the concept of a Floor Isolation System (FIS) with a TMD^34^. Feng and Mitta proposed the “mega subconfiguration” concept, aimed at creating usable interior substructures within the building and either have them vibrate in shear with diagonal fluid viscous dampers (FVDs) or tuned to vibrate as a conventional TMD^35^. However, as the mass ratio increases beyond the levels typically used in conventional TMDs, the sensitivity to frequency or damping detuning reduces (Fig. 1d, e)^29^. This simple fundamental analogy highlights the underlying merits of developing innovative damping solutions based on large-mass mobilisation.

In light of the above discussion, and with the aim of developing a large-mass response-reduction approach, inspiration was initially drawn from the architecture of traditional Japanese temples, in which different components, within the *Kumimono*, dissipate seismic energy by experiencing small differential motions and rotations relative to each other, only restrained by the *Shinbashira (*or central timber pillar), which has a further energy dissipation mechanism sliding and lifting over the *Shinso* (or heart stone) as shown in Fig. 1f^46^. The same behaviour can be extrapolated to a contemporary tall building, while exploiting the large inherent mass present. Considerable levels of damping could potentially be generated by allowing a significant portion of the building's own mass to move relative to the lateral stability system, while being connected together through springs and fluid viscous dampers (FVDs). The FVDs ensure that accelerations within the movable portion remain within comfort levels^13^, thus enabling a usable part of the building to be mobilised as a damper mass. The spring stiffness is not tuned to match optimal tuning as an orthodox TMD, but to resist the static component of the wind load and control differential displacements within serviceability conditions. Rather than adding extra mass, as in a conventional TMD, the proposed concept capitalises on the large inherent mass present in tall buildings and the large displacements they can experience at the top under service conditions to convert them into a design asset. This allows floors in tall buildings to experience minimal differential displacements with respect to the lateral stability system to provide substantially enhanced dynamic performance.

## Results

### Practical implementation

**Fig. 2:**
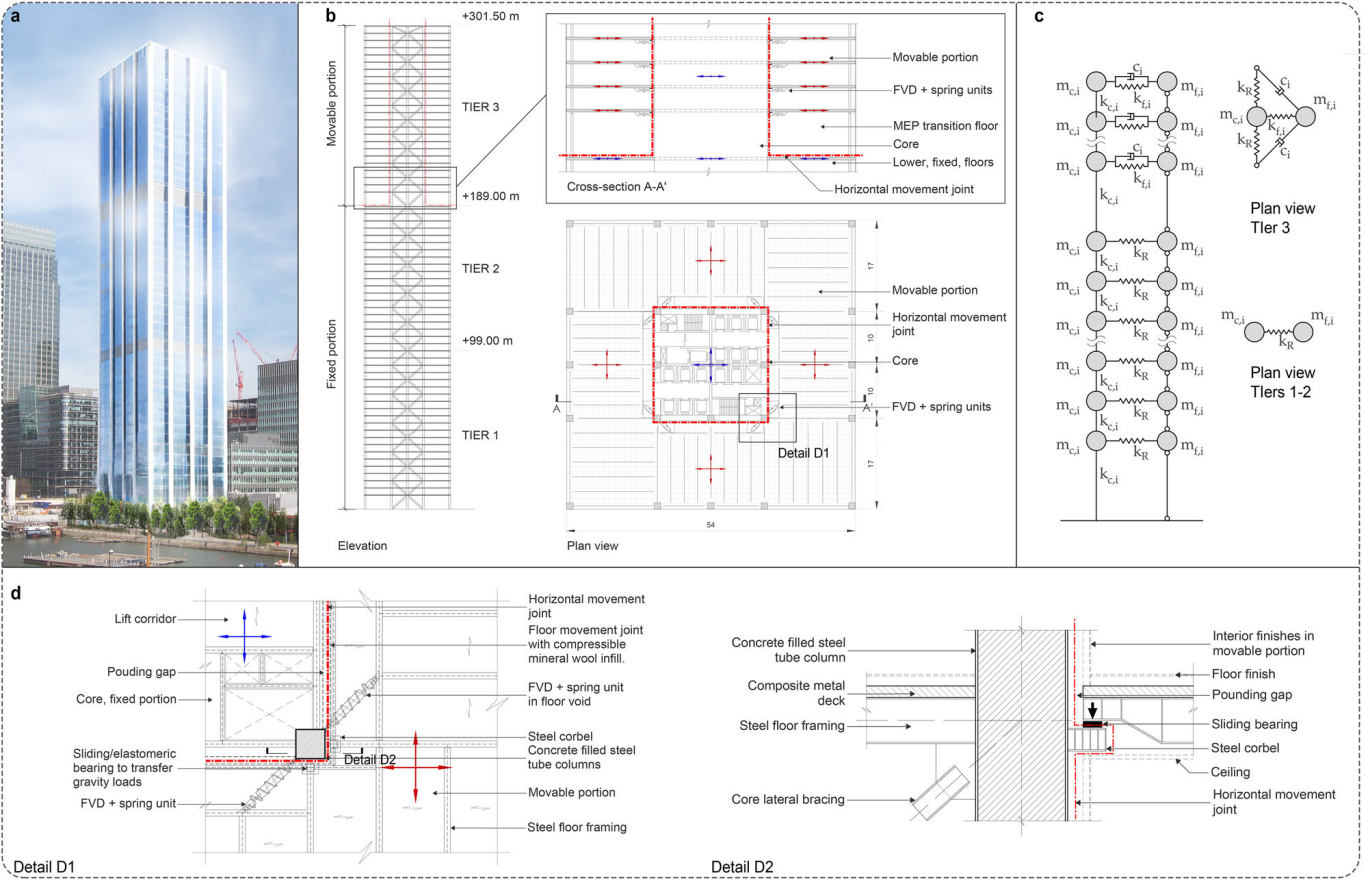
Prototype building definition. **a** 3d visualisation of the prototype building. **b** Elevation, plan and detail cross-section of the building at the movable interface. **c** Simplified lumped mass finite element model representation of the prototype building for dynamic analysis. **d** Interface implementation details.

The proposed approach was considered within an illustrative 65-floor, 300 m tall composite building, featuring a steel-braced central core (Fig. 2)^47^, and designed to Eurocode provisions^48,49^ for a considered basic wind speed of 21.5 m/s. The first two vibration modes of the building are translational with a period of 9.9 s, while the third mode is torsional with a period of 5 s and with no coupling to the first two modes. The initial design did not satisfy the wind-induced serviceability acceleration^14^, making it a candidate to explore a response-control mechanism.

Part of the own mass of the building was mobilised as a damper mass by allowing the floors to move from the core along the top third by providing a horizontal movement joint at floor level (Fig. 2b, d). While the study also investigates the option of mobilising the top sixth of the building, the presence of the plant floor at two-thirds of the height creates a natural position to accommodate the larger inter-storey drifts associated with the transition floor. The spring stiffness was set to allow a maximum differential displacement of 150 mm under the 50-year Eurocode 1 wind applied as a static load^49^, as a representative first value for preliminary design purposes. The building response was then examined in SAP2000^50^ using a multi-degree-of-freedom (MDOF) lumped mass model incorporating the mass and stiffness of the core and the individual floors with their respective stiffnesses (Supplementary Table 1). Office floor gravity loads are supported by the perimeter columns idealised as nominally pinned members, with the floor masses linked through rigid diaphragm constraints, consistent with the prototype modelling assumptions (Fig. 2c)^47^. Using the half-power bandwidth method, the equivalent modal damping was found to increase from the intrinsic value of 0.8% to about 6% with the proposed arrangement. This level far exceeds the 0.5%–2% typically attainable with conventional TMDs and common damping arrangements^18,31^.

Implementation is achieved by treating the interface between the mobilised floors and the core as a conventional movement joint sized to the design relative displacement demand. At floor level, standard movement joint assemblies, such as cover plates and fire-rated compressible infills, preserve compartmentation while permitting the required motion. Gravity loads are transferred to the core by connecting the floor framing to perimeter corbels with sliding joints, or elastomeric bearings, designed to accommodate the maximum expected movements (Fig. 2d). The helicoidal springs around the damper units ensure a robust lifecycle and provide lateral stability at both service and ultimate conditions. MEP systems crossing the interface are designed using typical flexible couplings and expansion loops according to conventional high-rise detailing practices, typically employed to accommodate thermal movements, and without the need to incorporate any bespoke components.

### Numerical assessment

**Fig. 3:**
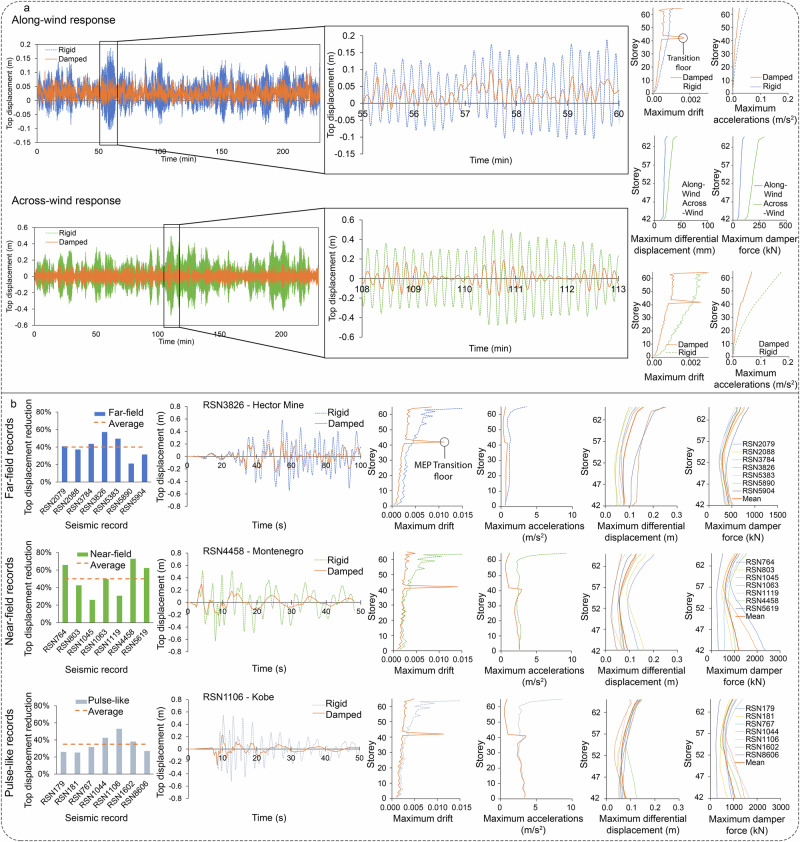
Numerical response of a decoupled building. **a** Along and across-wind response of a damped or decoupled building in comparison to a conventionally rigid design in terms of top displacement, inter-storey drifts, accelerations, differential displacements, and damper force. **b** Seismic response to three sets of seven far-field, near-field, and pulse-like real earthquake records assessed in terms of top displacement, inter-storey drifts, accelerations, differential displacements, and damper force with the records denoted as per their reference in the PEER NGA database (Supplementary Table 2)^65^.

The numerical simulations described in this section provide more detailed insights into the dynamic response under realistic wind and seismic scenarios using the MDOF lumped-mass model (Fig. 3) (with further information given in the “Methods” section). These studies also provide additional insights to steer subsequent experimental assessments and further illustrate the benefits of decoupling a usable portion of the building and mobilising its own mass as a damper mass. Firstly, under along- and across-wind time histories (Supplementary Fig. 1a), the decoupled damped system showed significant enhancement in performance when compared to a rigid counterpart (Fig. 3a). Since structural sizes in tall buildings are usually determined by wind demands, this is a key differentiator that could translate to large savings in material use. Reductions attained in peak top displacements were up to 41% and 60% in the along- and across-wind directions, respectively; reductions in average inter-storey drifts were up to 31% and 53%, respectively; and in average floor accelerations by 52% and 62%, respectively. The mean peak differential displacements between the floor and the core at the decoupled levels remained within magnitudes that can be detailed using conventional approaches, at 33 and 58 mm in the along-wind and across-wind directions, respectively. The peak damper forces were also low, at 70 and 188 kN on average, in the along and across-wind directions, respectively.

Although tall building design is typically governed by wind, ensuring seismic resilience after frequent events and life-safety under rare earthquakes is often necessary. The seismic performance of the building was therefore also assessed using three sets of seven real earthquake records, scaled to an assumed peak ground acceleration of 0.25 g (Supplementary Table 2, Supplementary Fig. 1b). The records covered far-field, near-field, and pulse-like types, noting that conventional mass damping systems are typically ineffective for pulse-like records^32^. The results demonstrated the ability of the proposed approach to drastically improve the seismic response (Fig. 3b). When compared to a rigid counterpart, on average, top displacements decreased by 42%, inter-storey drifts by 30%, and accelerations by 34%. As the damped floors are dynamically decoupled from the base excitation, the movable tier showed more pronounced improvements, with inter-storey drifts reducing by 52% and accelerations by up to 74% when averaged over the decoupled floors. While this behaviour somewhat generally resembles that of base-isolated buildings, albeit these are ineffective in tall buildings due to their higher loads and longer first natural period, the response control mechanism also has a reciprocal beneficial effect in the remaining structure, reducing overall demands. The different behaviour of the lower, fixed floors and the movable portion is evident at the transition floor. The peak inter-storey drift at this level remains below the maximum inter-storey drift observed at the top of the building in all conditions. It also remains below the ultimate drift limit of 0.03^51^, which can be readily accommodated within the louvred façade of the MEP floor using conventional detailing practices. The mean of the peak differential displacements remained low, at 83 mm, with a damper force of 668 kN, significantly larger than for wind, due to the higher frequency content. Although the level of relative displacements largely depends on the site seismicity, for areas of high seismicity, it is expected that the system would be subjected to larger differential displacements under moderate and extreme events. Accordingly, while it is common practice to accommodate damage in finishes and MEP services at those levels, the movement joint gap should be designed to accommodate the displacements expected from the Risk-Targeted Maximum Considered Earthquake (MCE_R_) without pounding, and to avoid disproportionate collapse in all instances.

The results highlight the potential of the system in enhancing tall building safety, efficiency and resilience, significantly reducing non-structural damage and downtime under both frequent and large wind and seismic events. Furthermore, the results also illustrate the combined energy dissipation mechanism of the system. For long-period content, such as wind or far-field records, around the first building vibration period, the decoupled mass mitigates overall response by behaving akin to a detuned mass damper, with a slight phase offset, reducing generalised forces with a minimal stroke. For short excitation periods, such as for near-field or pulse-like records, which engage the higher vibration modes, energy is dissipated through the differential motions at both ends of the dampers, resembling the behaviour of a distributed damping system. This dual action, enabled by the large mobilised mass, explains the observed broadband effectiveness and tolerance to detuning across both wind and seismic excitations.

### Experimental methodology

Due to the novelty of the concept, experimental verification was essential. Physical evaluation of the approach under wind loading was, however, particularly challenging. Tall building wind response is usually assessed using high-frequency force balance (HFFB) or high-frequency pressure integration (HFPI) tests, but these methods cannot capture the non-classical damping configuration nor the interaction of the movable portion with the wind flow. These features, together with the lack of precedent, required the use of a fully aeroelastic model, also precluding the use of uncalibrated Computational Fluid Dynamics (CFD) approaches^52–55^.

For the experimental assessment, a 1:300 scale MDOF aeroelastic model was designed to reproduce the aerodynamic response of the prototype building under realistic wind scenarios, with dynamic matching enforced through scaling and similarity ratios^56,57^ (Table 1). The complexity and sensitivity of aeroelastic models, which restrict their use to specialised research applications, together with the need to incorporate the decoupled mass explicitly, necessitated the development of a parametric framework for aeroelastic model generation. A finite element digital twin of the physical model was implemented in Oasys GSA^58^ and controlled from within the Grasshopper environment^59^, enabling full parametric definition of geometry, section properties and mass distribution. This digital twin was coupled with a genetic algorithm optimisation solver^60^, which iteratively adjusted member sizes and mass distribution to match, within fabrication constraints, the target scaled frequencies, mode shapes and scaled mass and stiffness distributions of the prototype.

**Table 1:** Similarity ratios for aeroelastic model parameters

Model parameter	Similarity parameter	Scale ratio
Length	${{\rm{\lambda }}}_{L}$	1/300
Velocity	${{\rm{\lambda }}}_{V}$	1/4
Time	${{\rm{\lambda }}}_{{\rm{T}}}=\frac{{{\rm{\lambda }}}_{L}}{{{\rm{\lambda }}}_{{\rm{V}}}}$	1/75
Acceleration	${{\rm{\lambda }}}_{{\rm{\alpha }}}=\frac{{{\rm{\lambda }}}_{V}^{2}}{{{\rm{\lambda }}}_{{\rm{L}}}}$	18.75
Damping ratio	${{\rm{\lambda }}}_{\zeta }$	1
Density	${{\rm{\lambda }}}_{\rho }$	1
Mass	${{\rm{\lambda }}}_{{\rm{M}}}={{\rm{\lambda }}}_{\rho }{{\rm{\lambda }}}_{L}^{3}$	1/2.7 × 10^7^
Frequency	${{\rm{\lambda }}}_{{\rm{f}}}=\frac{{{\rm{\lambda }}}_{V}}{{{\rm{\lambda }}}_{L}}$	75
Force	${{\rm{\lambda }}}_{F}={{\rm{\lambda }}}_{\rho }{\left({{\rm{\lambda }}}_{L}{{\rm{\lambda }}}_{V}\right)}^{2}$	1/1.44 × 10^6^
Bending moment	${{\rm{\lambda }}}_{{\rm{BM}}}={{\rm{\lambda }}}_{\rho }\,{{\rm{\lambda }}}_{V}^{2}\,{{\rm{\lambda }}}_{L}^{3}$	1/4.32 × 10^8^
Stiffness	${{\rm{\lambda }}}_{K}={{\rm{\lambda }}}_{\rho }\,{{\rm{\lambda }}}_{V}^{2}\,{{\rm{\lambda }}}_{L}$	1/4800
Damper coefficient	${{\rm{\lambda }}}_{C}={{\rm{\lambda }}}_{\rho }\,{{\rm{\lambda }}}_{V}^{2}\,{{\rm{\lambda }}}_{L}\,{{\rm{\lambda }}}_{T}$	1/3.6 × 10^5^

**Fig. 4:**
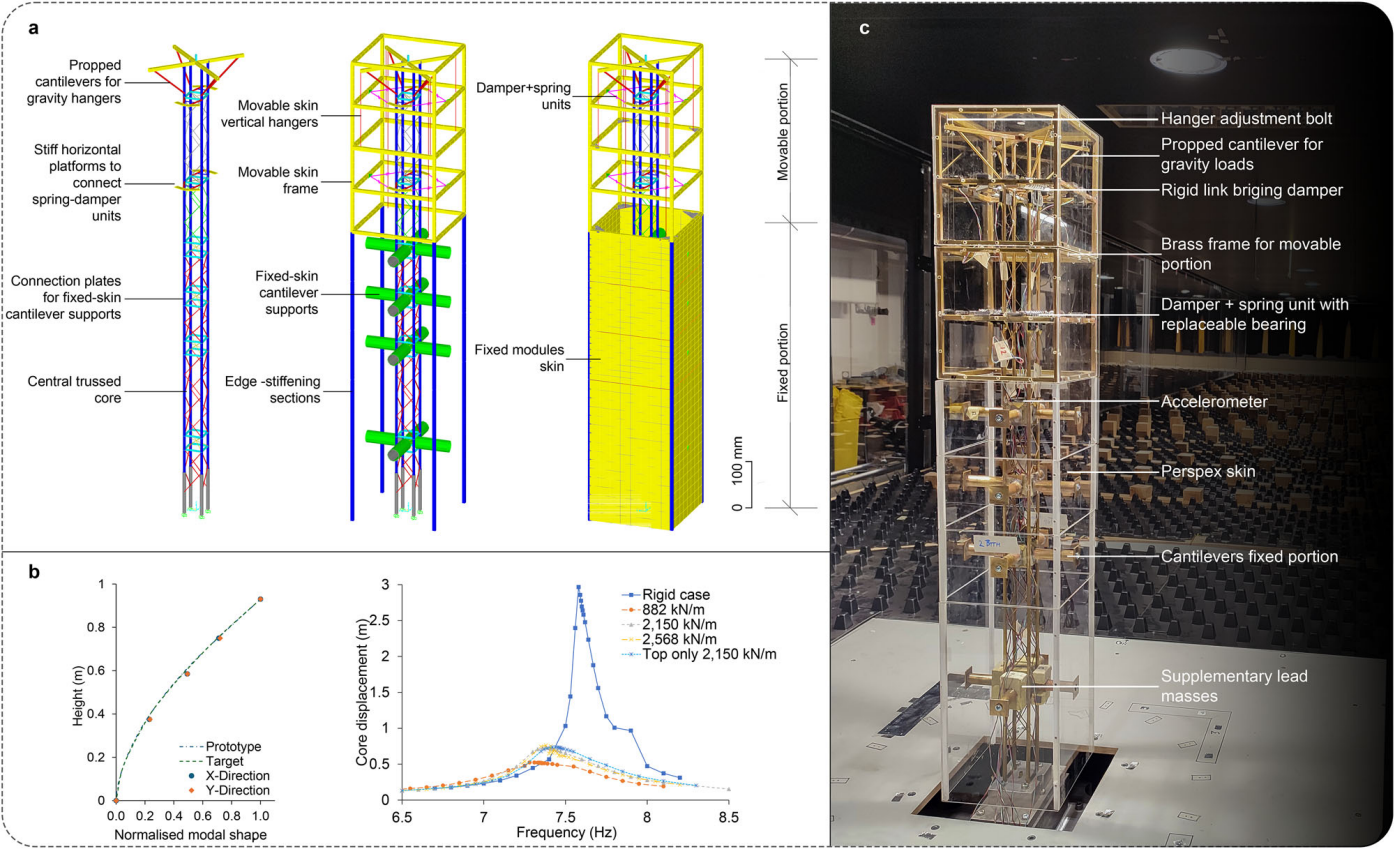
Aeroelastic model definition. **a** Finite element representation of the scaled physical model at different fabrication stages, from left to right: central core, core with outer frame, and core with lower skin. **b** Forced vibration results with mode shape comparison and time history of the normalised displacement at the top of the tower under free vibration in both directions. **c** View of the MDOF aeroelastic model in the wind tunnel test.

The aeroelastic model was discretised into six nominal degrees of freedom, each representing the mass and stiffness of a vertical segment of the tower. The top two segments, corresponding to 12 floors each, were allowed to move horizontally relative to the central core (Supplementary Table 3). Miniature calibrated springs and fluid viscous dampers connected the outer “skin”, representing the upper office floors, to the core (Fig. 4a). The spring–damper assemblies were interchangeable to allow three different representative stiffness values (0.78 N/mm, 1.9 N/mm and 2.27 N/mm at model scale). These correspond to horizontal stiffnesses and differential displacement under the EN 1991-1-4 static wind load per floor at the prototype scale of 882 kN/m and 410 mm, 2150 kN/m and 170 mm, and 2568 kN/m and 140 mm, respectively) and the locking of one or both movable modules, enabling direct comparison with a rigid counterpart. The optimal spring stiffness for the system would correspond to that where the frequency of the mobilised mass is tuned to match the frequency of the building, as a conventional TMD. However, this is not generally feasible due to its low value, as it would result in unacceptable relative movements. Accordingly, every specific application requires calibration of the lower spring stiffness and corresponding maximum relative displacements that can be realistically incorporated.

The final configuration was tuned to reproduce the first two translational modes and validated through free-vibration tests, achieving a Modal Assurance Criterion (MAC) of 0.999 for both translational mode shapes and close agreement with the target natural frequencies and intrinsic damping levels (Fig. 4b, Table 2). Beyond its direct application in this study, this confirms the fidelity of the parametric model-generation framework and its potential to streamline the development of advanced aeroelastic models for tall buildings (Fig. 4c).

**Table 2:** Modal parameters of the prototype building, FEM digital twin-model, and MDOF aeroelastic model with and without base load cell

Mode number	Type	Scaled Freq. of prototype building	Freq. of FEM digital twin-model	Freq. MDOF aeroelastic model on rigid base	Freq. MDOF aeroelastic model on load cell	Damping ratio MDOF aeroelastic model
1	Trans. X	7.5 Hz	7.5 Hz	7.5 Hz	7.3 Hz	0.4%
2	Trans. Y	7.5 Hz	7.5 Hz	7.70 Hz	7.3 Hz	0.6%
3	Torsion	14.9 Hz	11.3 Hz	10.1 Hz	10.1 Hz	−

**Fig. 5:**
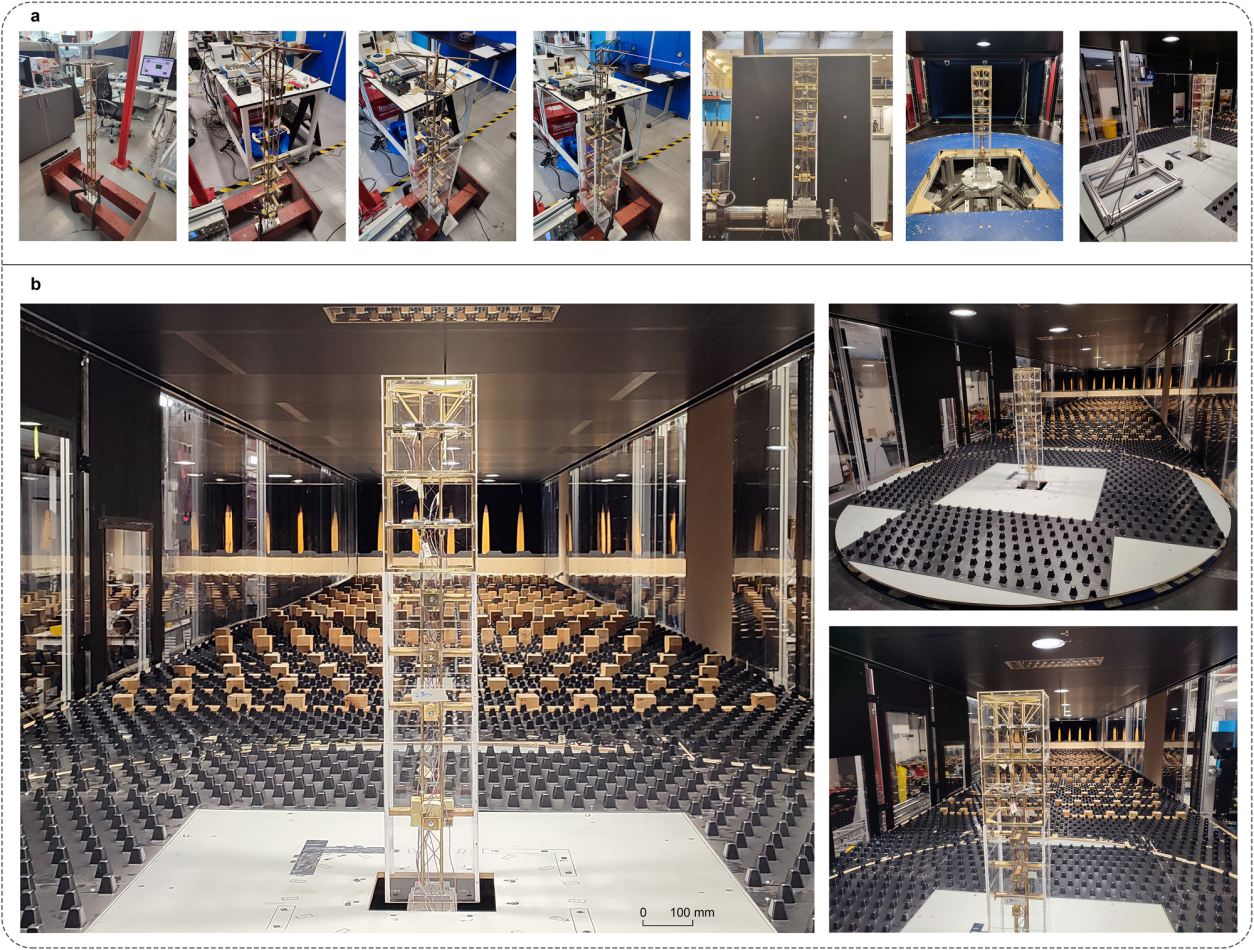
Aeroelastic wind tunnel test model. **a** MDOF aeroelastic model testing at different stages during construction, under free and forced vibration, and in the wind tunnel. **b** MDOF aeroelastic model in the wind tunnel.

Physical model verification was further complemented prior to wind tunnel testing with a series of dynamic seismic tests, including harmonic and scaled seismic excitations. The capacity of the dynamically-scaled physical model to generate large levels of damping was verified under forced vibration, with a significant reduction in resonant peak amplitude when compared to a rigid counterpart (Fig. 4b). The complementary seismic tests showed consistent results with the numerical predictions^61^ (Fig. 5a)

The boundary layer wind tunnel (Fig. 5b) was configured using an assumed terrain analysis with speeds at the top of the model ranging from 22 m/s (below the 1-year return period wind speed) to 50 m/s (33% higher than the ultimate strength demand), above which model integrity could be compromised. Testing included a speed sweep for all stiffness configurations at 0°, as the most critical direction, and a full 360° sweep for the rigid and the mid-stiffness (2150 kN/m) damped cases. The model was equipped with 9 miniature dual-axis accelerometers at multiple levels and a 6-axis high-precision load cell (Supplementary Fig. 2). All results are shown in unscaled prototype units for ease of reference. The X-direction is set at 0°, in the direction of the incoming wind, and the Y-direction transversely, at 90°. Additional information on the formulation, fabrication, and validation of the aeroelastic model and wind field simulation can be found in the “Methods” section.

### Aeroelastic wind tunnel results

**Fig. 6:**
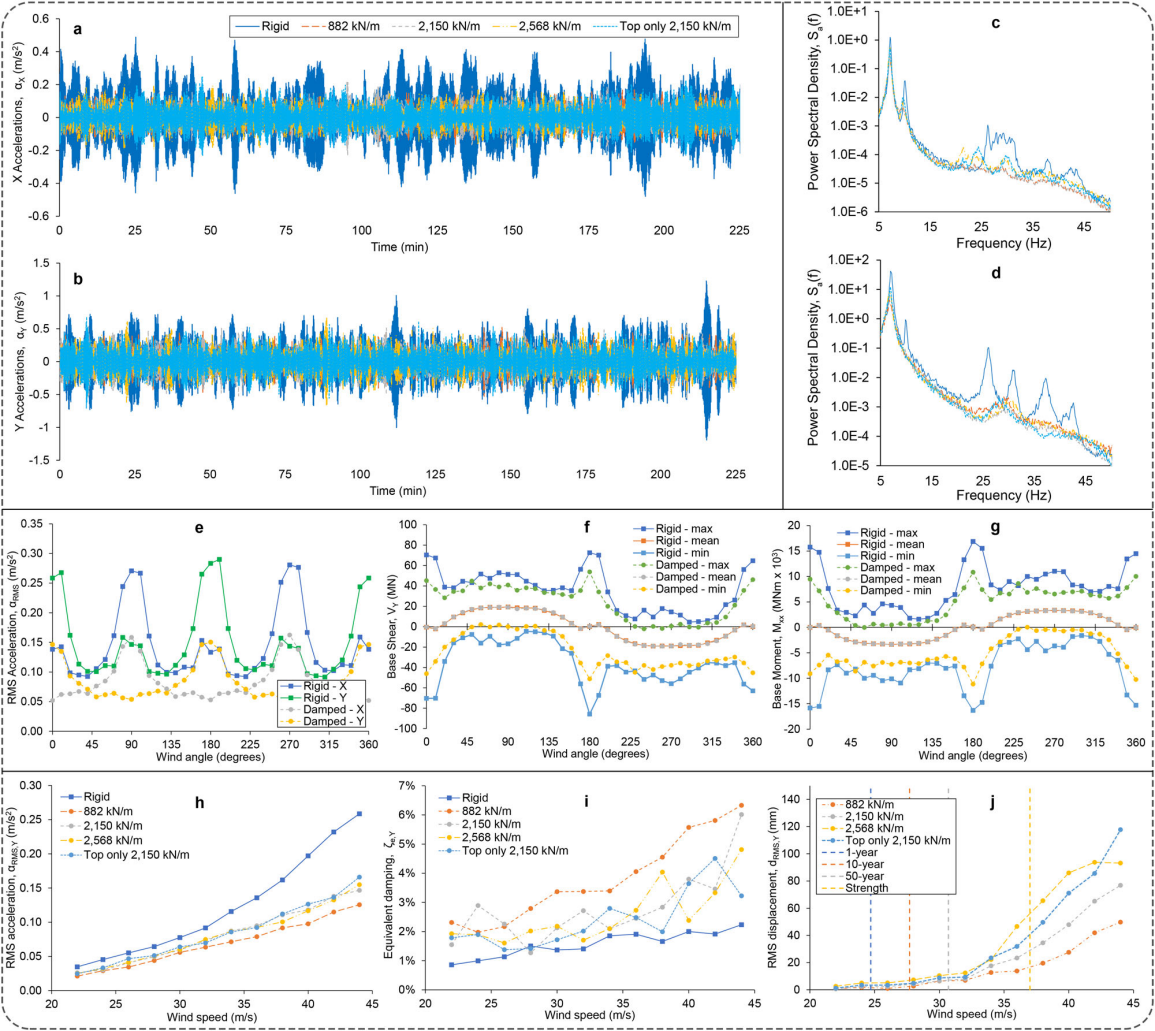
Aeroelastic wind tunnel test results. Acceleration time-history response for 0° wind direction at 44 m/s in, **a**, Along-wind direction, and **b** Across-wind direction. Power Spectral Density at 0° wind direction at: **c** 22 m/s and **d** 44 m/s wind speeds. Rigid and damped baseline case response for varying wind angle at 44 m/s for, **e** RMS top core acceleration response; **f** Base shear in the X-direction; and, **g** Base overturning moment in the Y-direction. Response of the five tested configurations with varying wind speed at 0° wind direction in: **h** RMS top core accelerations in the Y-direction; **i** Equivalent first mode damping in the Y-direction; and **j** RMS differential displacements at the top module.

The wind tunnel test results validated the superior performance of all damped configurations compared to the conventional rigid counterpart. The acceleration time-histories of the four damped configurations in the along-wind (Fig. 6a) and across-wind directions (Fig. 6b) exhibit peak acceleration reductions of up to 71% and 58%, respectively (Supplementary Movie 1). The response shows similar trends as in the numerical assessments, with significant mitigation of the resonant vibration peaks. The effectiveness of the system is also evident through the reduced energy content of the Power Spectral Density response functions at 22 m/s (Fig. 6c) and 44 m/s (Fig. 6d).

As expected, the building shows a more pronounced response in the across-wind direction when subjected to winds perpendicular to one of the sides, which also indicates the enhanced effectiveness of the decoupled arrangement in mitigating the resonant vibration (Fig. 6e). Strength demands such as base shear (Fig. 6f) and overturning moment (Fig. 6g) show a similar trend, with peak values reduced by up to 25% and 36%, respectively, for the mid-stiffness spring, and reducing up to 46% and 50%, respectively, for the soft, 882 kN/m per floor, stiffness case.

The benefits of the decoupled arrangement can be further ascertained by assessing the response for varying wind speeds at the critical wind direction of 0°. Focusing on the across-wind as the direction with the largest response amplitude, RMS accelerations show a consistent response reduction even for low wind speeds (Fig. 6h), by up to 68% in the along-wind direction and 51% in the across-wind direction.

The results also illustrate the significant levels of response reduction that can be achieved even if only the top 12 floors are mobilised. Using the Time Domain Decomposition technique^62,63^, the ability of the system to generate up to 6.3% equivalent damping is verified (Fig. 6i). By assessing the level of differential displacement between the core and the floors, it is evident that the performance benefits are achieved whilst exhibiting minimal displacement levels. The maximum and RMS differential displacements remain below 50 mm for the 10- and 50-year wind events, with only the ultimate strength combinations marginally exceeding this (Fig. 6j). Interestingly, the soft, 882 kN/m floor stiffness configuration gives the lowest differential displacement, due to its enhanced vibration control that results in a lower vibration amplitude. The small service-level relative movements can be accommodated in the services crossing the core-to-floor interface, using conventional high-rise flexible connections and thermal expansion details. Vertical transportation can remain fully contained within the core, and fire-rated movement joint assemblies bridge over the gap, maintaining fire compartmentation between levels.

Overall, the experimental results confirm that mobilising a portion of the building's own mass can deliver significant, reliable levels of damping. Across all stiffness configurations and wind speeds, the decoupled arrangement reduced accelerations, base shear and overturning moments relative to the rigid counterpart, while maintaining differential displacements within serviceability limits for conventional MEP, fire, and finishes detailing. Together with the numerical assessments, these findings demonstrate that the concept is mechanically robust, practically realisable and capable of substantially reducing both serviceability- and strength-level demands making the most of the mass and movements inherently present in a building. This opens the door for lower-carbon, high-performance tall building designs with enhanced safety and resilience, which can contribute to more sustainable and disaster-resistant urban development.

## Discussion

This study challenges the traditional premise of conceiving tall buildings as relatively rigid, static entities and proposes a paradigm of accepting and harnessing movements. As established by the detailed numerical and experimental assessments, mobilising a portion of a building own mass and decoupling it from the rest of the structure can substantially reduce the demands on the superstructure and the foundations, enabling direct material reductions with cost and carbon benefits. The results demonstrate compelling reductions in both serviceability and strength-level demands. Paired with the use of conventional spring-damper technology, the feasibility of implementing such an approach in tall buildings with current design and construction workflows is confirmed. This offers the potential for more efficient and resilient tall buildings that can safely and economically support global urban growth in the transition towards a resilient Net Zero built environment. Although this study focuses on tall buildings, the applicability of the proposed concept or its variations can also be explored for long-span bridges, offshore structures, wind turbines or slender communication towers.

## Methods

### Numerical assessments under wind conditions

The 300 m central core building was modelled within the finite element software SAP2000^50^ using an MDOF representation where the mass of every floor at the core and the office floor were represented as independent single degrees of freedom. The simplified lumped mass model was benchmarked to the detailed 3D model of the building, replicating the total mass and mass distribution, and achieving a close match between the first three translational modal periods^47^. Vertical gravity loads at the office floors are transferred through the perimeter column line, which is idealised as nominally pinned (gravity-only) members, with floor masses coupled via rigid links and springs and dampers, as appropriate, to represent the effect of the system while avoiding unintended participation of the gravity system in the lateral stiffness, consistent with the design of the prototype building. The dynamic properties of the prototype building and lumped mass model are shown in Supplementary Table 1. Within the model, the damper elements were represented following the Maxwell model of viscoelasticity, with a linear spring in series with a nonlinear dashpot to capture the effect of the damper and brace stiffness in the formulation, and a relaxation time constant of 0.01. Due to the non-classical damping arrangement of the system and the FVD's velocity-dependency, the system response was investigated using nonlinear time-history analysis with direct integration. Additional information on the numerical idealisation can be found elsewhere^47^.

The 300 m central core building was subjected to a pair of wind tunnel time-history forcing functions consisting of an along-wind and an across-wind time history record for a 10-min mean wind speed of 24.5 m/s at 10 m, and an hourly mean of 40.8 m/s at 600 m. This corresponds to a wind event for a 50-year return period with hurricane conditions with an upwind suburban terrain around the 360 degrees of the building, and a wind climate model based on local surface wind measurements at Taipei Songshan Airport, with the corresponding wind profile for Taipei as stipulated in the Taiwan Building Code 2007 (Supplementary Fig. 1a). To adapt to the different building dimensions and speed, the wind force was proportionally increased to match the slightly increased prototype building width and peak amplitude to match a top displacement of *H*/500 as the design target. The decoupled arrangement was investigated using a static stiffness defined to enable a maximum differential displacement of up to 150 mm under the EN 1991-1-4 50-year static wind load^49^ and with an optimal damper coefficient to maximise first mode damping for the selected stiffness. The response was evaluated by comparing a damped or decoupled arrangement with a conventionally rigid counterpart in terms of five performance indices, namely: top displacement, floor-by-floor accelerations, inter-story drifts, damper forces and differential displacements, as the key indices describing tall building response and directly related to the required structural sizes.

### Numerical assessments under seismic conditions

The response of a prototype decoupled building was evaluated under realistic earthquake loading using three sets of seven real earthquake acceleration-time histories. These were selected to correspond to seven far-field records, with a distance from the fault beyond 200 km; seven near-field records, with a distance of less than 20 km; and seven pulse-like records, as defined in Chang et al^64^. All records were extracted from the PEER NGA Database^65^, to match the EC8 Type 1, Soil C, target spectrum with a PGA of 0.25g^66^, minimising the mean squared error (MSE) over a period from 2 s to 10 s with scaling factors ranging from 0.2–5.0^67^. The records were selected with a magnitude, *M*, of 6.5–7.3, and a shear wave velocity, *V*_*s*_, from 180 m/s to 360 m/s. Each record is referred to by its number in the PEER NGA Database, with the seismological data, scaling factors, and corresponding MSE values given in Supplementary Table 2. The unscaled acceleration-time histories and scaled response spectra are shown in Supplementary Fig. 1b–d, for far-field, near-field, and pure pulse-like, respectively.

### Aeroelastic model formulation and design

Aeroelastic models are generally categorised as either single-degree-of-freedom (SDOF) or multi-degree-of-freedom (MDOF). MDOF models can simulate the characteristics of the several modes and give a closer response to that of the prototype building. Except for special applications such as chimneys, transmission towers, or others that can be represented as a scaled continuous model^68,69^, buildings can generally be represented using a reduced DOF lumped-mass approximation of the original structure^54,70^. In such models, the design aims to closely replicate the dynamic behaviour of the prototype by matching key characteristics such as modal properties, mass distribution, length scale, damping, and Strouhal number. However, it is important to note that fully satisfying all similarity laws in practice is impossible. Similarity in non-dimensional parameters such as the Reynolds or Froude numbers is shown to be impractical at the test scale. Also, since it has a limited influence for wind tunnel testing of non-circular cross-sectional buildings and for vibrations in the horizontal direction against the gravity vertical direction, respectively, it can generally be neglected^71,72^.

For the definition of the aeroelastic model, the geometric scale, *λ*_*L*_, was defined as 1/300 to ensure the model projection satisfies the 5% wind tunnel tests blockage ratio while aligning the top of the model to the Pitot-static probe that, 4 m away from the turntable centre, measures the wind velocity in the tunnel and ensures enough space to incorporate the miniature spring and dampers in the model. The velocity scale, *λ*_*V*_, was set to 1/4 to ensure that the natural frequency of the first mode is below 10 Hz, with a frequency scaling factor, *λ*_*f*_, of 1/75, to ensure that the model vibrates within the low-frequency range and thus avoids compromising the model integrity due to violent shaking. The air density ratio, *λ*_*ρ*_, was defined as 1.0. Once these have been defined, all remaining similarity ratios can be derived as shown in Table 1.

The dynamic similarity between the prototype and the MDOF aeroelastic model is achieved by matching their generalised properties shown in Eqs. 1–5, for every mode of interest, *n*, where *i* corresponds to the prototype floor number and *j* to the corresponding aeroelastic model module within which it is grouped^56,73^.

1 $${\omega }_{{model}}={{\rm{\lambda }}}_{{\rm{f}}}\,{\omega }_{{prototype}}$$

2 $$\,{\zeta }_{{model}}={\zeta }_{{prototype}}$$

3 $${{m}_{j}\varphi }_{{nj}}^{2}=\sum _{i={i}_{1}}{{\rm{\lambda }}}_{{\rm{m}}}{m}_{i}{\varphi }_{{ni}}^{2}$$

4 $${{k}_{j}\triangle \varphi }_{{nj}}=\mathop{\sum }\limits_{i={i}_{1}}^{{i}_{2}}{{{\rm{\lambda }}}_{{\rm{K}}}k}_{i}{\triangle \varphi }_{{ni}}$$

5 $$\,{I}_{j}{\varphi }_{n\theta j}^{2}=\mathop{\sum }\limits_{i={i}_{1}}^{{i}_{2}}{{\rm{\lambda }}}_{{\rm{I}}}{I}_{i}{\varphi }_{n\theta i}^{2}$$

in which *m*, *k*, and *I* are, respectively, the mass, stiffness, and rotational mass, and ${\triangle \varphi }_{{nj}}={\varphi }_{{ni}}-{\varphi }_{{ni}-1}$ and ${\triangle \varphi }_{{nj}}={\varphi }_{{nj}}-{\varphi }_{{nj}-1}$.

Due to the complexity of the model, arising from the aim to explicitly incorporate the damping system, with a portion of the model defined as movable and connected with miniature springs and dampers, the formulation of Eqs. 1–5 is more involved than for conventional aeroelastic models. To ensure model accuracy while enabling design iterations, a digital-twin finite element model of the MDOF aeroelastic model was created parametrically in the software Oasys GSA^58^ using the new GSA-GH component^74^ in the visual programming interface Grasshopper^59^. As GSA-GH can run within the Grasshopper environment, it was thus possible to automatically extract the model frequencies, nodal masses, and modal shapes for every run and, by linking them with Eqs. 1–5, assess the suitability of every configuration. By combining the results into a weighted fitness function, where the target modes and parameters were assigned an importance factor based on their relevance to the end results. The multi-variable genetic algorithm optimisation solver Galapagos^60^ was then used to iterate between thousands of potential model configurations to find the closest fit to the specified target behaviour once practical considerations are incorporated into the design. The optimisation algorithm was set to prioritise matching the response under the first two translational modes, frequencies, mode shape, and mass distribution, followed by the second translational modes and having the torsional mode as a third priority, yet ensuring this remained the third mode with no coupling. The complete Grasshopper script and defined fitness function can be found within the Supplementary Material provided with this paper.

The optimum vertical grouping split and number from the prototype to the reduced DOF of the model was calculated using the same genetic algorithm solver. The aim was to find the combination that matched the first mode mass distribution as per Eq. 3 and reflected, as closely as possible, the second translational mode. Based on the results of the study, it was decided to discretise the model into 6 modules or nominal DOFs. This resulted in a bottom module, capturing the lower 20 floors, three intermediate modules, each representing 7 prototype floors, and two movable modules at the top incorporating 12 stories each (Supplementary Table 3).

The final aeroelastic model consists of a brass central core, four fixed facade modules that cantilever from the central core, and two top movable modules that hang from a propped cantilever that extends from the core, as illustrated in Fig. 4. Suspending the movable modules provides the required vertical support while allowing the spring–damper units to govern the horizontal coupling. This avoids parasitic friction and inadvertent outrigger effects that would be difficult to control at a 1:300 scale and could otherwise alter the intended modal properties and energy dissipation mechanism. The core was made from four circular hollow sections that run continuously throughout the height, rigidly joined by horizontal hollow sections at node levels with solid diagonals in between, forming a trussed core that provides lateral stability to the tower. The different core elements and vertical setting-out vary in size and thickness throughout the height, as reported by the genetic algorithm solver to match the target behaviour. The model skin was formed from transparent Perspex sheets 1.5 mm thick, bolted to a supporting frame with countersunk bolts and separated with a 1.5 mm gap between modules to avoid contact when vibrating at large amplitudes. The top two movable blocks were connected at node levels via 4 miniature springs and damper arrangements set out at 45°, to match the prototype building arrangement, which extends from a horizontal platform designed to be horizontally stiff but not carry any vertical load. The damper and spring arrangement was designed as interchangeable to test different configurations, including replacement by a rigid bar to enable comparison with a conventional rigid building. The model was anchored at the base to a solid steel plate that links it to a load cell and the rigid support frame under the wind tunnel.

The accuracy of the aeroelastic model and its ability to replicate the same behaviour as the prototype building when incorporating the IDS were also checked before fabrication using the finite element package SAP2000^50^ to run non-linear time history analysis. Using the scaled damper and spring coefficients, the aeroelastic model was verified to match the 6% additional equivalent damping shown in the numerical studies on the prototype building.

### Model fabrication, instrumentation and validation

The intricate requirements of this study necessitated an even more controlled fabrication process beyond that already present in common aeroelastic models, with large sensitivity to any variations in mass or stiffness. The key structural elements were tested to verify their actual stiffness and elastic modulus, and the weight of every miscellaneous element introduced into the model was verified to accurately account for its contribution. Moreover, in order to ensure an accurate final product and the ability to identify any potential deviation, the model behaviour was tested at key assembly stages and compared to that of an equivalent finite element model at the same stage (Fig. 5a). By measuring the model response under free vibration in both principal directions, it was possible to verify the adequacy of the model and reassess the design when any minor deviation occurred as fabrication progressed (Fig. 5b).

To maximise the insights from the study, the model was designed to allow modifications to the spring-damper arrangement. By allowing the removal of the unit and replacement of the springs, it was possible to investigate the response under three different spring stiffnesses. These were defined to match target differential displacements under the EN 1991-1-4 50-year wind load^49^, applied as a static load for initial reference in preliminary design. The selected spring stiffness values were 0.78 N/mm, 1.9 N/mm, and 2.27 N/mm, corresponding to displacements of 410 mm, 170 mm, and 140 mm, respectively. The miniature dampers were selected as linear viscous dampers, ensuring negligible friction or static stiffness, and calibrated using an in-house set-up to meet the scaled damper coefficient target of 65 Ns/m.

The model response was captured with 9 miniature dual-axis accelerometers that were integrated into the aeroelastic model, featuring amplified analogue output and a sampling frequency of 2 kHz. These sensors were positioned to capture X and Y direction displacements at four critical levels: the torsional response of both the core and skin, overturning effects, and differential displacements between the core and skin. The placement and orientation of the accelerometers are illustrated in Supplementary Fig. 2a. The set-up also included a 6-axis high-precision load cell, ATI Delta IP 65 SI330-30, that was installed at the bottom of the model to capture the base reactions. While the presence of the load cell slightly reduced the model frequency due to its non-infinitely rigid nature, the insights that it would provide were determined to outweigh the very small frequency shift.

In addition to interim tests during construction, the modal parameters of the complete MDOF aeroelastic model were verified by performing a series of free vibration decay tests. In order to capture any secondary effects, the final set of tests was performed with the tower set in the wind tunnel and with the rigid link preventing any differential movement at the damper. As shown in Table 2, the aeroelastic model provided a near-perfect match to the target scaled properties of the prototype. The first natural frequencies of the model under a rigid base were 7.56 Hz in the X direction and 7.70 Hz in the Y direction, with only a 2% difference from the 7.5 Hz target frequency. Based on these results, it was decided not to alter the velocity scale. The only significant discrepancy can be observed for the torsional mode. This was because the top two movable blocks have a large portion of their mass around their perimeter, in the skin and its frame, and thus have a higher rotational inertia than the prototype.

From the results, it was possible to verify the accuracy of the model and its suitability for wind tunnel testing. The favourable results obtained also indicated the merits of the proposed parametric aeroelastic model generation procedure.

### Wind field simulation

The wind tunnel testing was conducted at the 10’ × 5’ Wind Tunnel Facility at Imperial College London (Fig. 5b). This closed-loop, temperature-controlled facility has a test section measuring 3.05 m in width, 1.52 m in height, and 20 m in length. The tunnel includes spires, fences, and surface roughness elements, which, combined with its long fetch, enable the development of a representative atmospheric boundary layer. A 3-metre diameter turntable is also located at the downstream end of the test section, providing a stiff support base for model mounting and orientation adjustments.

The wind simulation details were specified assuming a hypothetical, London-based terrain analysis, from the location of the tower outwards, following the ESDU Atmospheric Boundary Layer^75,76^. The wind characteristics were defined using values of *z*_*0*_ = 1 m for a fetch of 2 km, *z*_*0*_ = 0.5 m for the next 10 km, *z*_*0*_ = 0.3 m for the next 18 km, and *z*_*0*_ = 0.03 m further afield. The obtained target mean velocity, *U*, and turbulence intensity distribution profile, *I*_*u*_, were replicated at scale in the wind tunnel by adjusting the spires and surface roughness fetch. The measured data, superimposed on the ESDU and EN 1991-1-4 values^49^, over Terrain Category IV and *z*_*0*_ = 1 m, is shown in Supplementary Fig. 2b-B1. The measured pre-multiplied Power Spectral Density at model height along the best fit von Kármán distribution is shown in Supplementary Fig. 2b-B2.

The reference wind velocity, *U*_*ref*_, at which the results are presented, was set as the top of the building, *Z*_*ref*_, 300 m at unscaled height, and 1 m at the model scale, matching the wind tunnel Pitot-static probe that measures the wind speed throughout the test. The 50-year London wind base velocity, as per EN 1991-1-4^49^, set as 21.5 m/s, results in a wind speed of 30.7 m/s at the reference height. Extrapolating the calculated wind velocity to the 1 and 10 years return periods and the equivalent speed when calculating ultimate strength demands results in 24.7 m/s, 27.7 m/s, and 37.4 m/s at the reference height.

The MDOF aeroelastic model, as shown in Fig. 4c, was positioned at the centre of the 3 m turntable, with the immediate surroundings of the tower maintained as generic roughness to ensure the results could be extrapolated for general applications. The wind response was analysed over the full 360° range, accounting for the tower symmetry. This was performed by assessing the response at 10° intervals from 0° to 90°, and subsequent tests at 45°, 135°, 180° and 270° to provide verification points. The full 360° characterisation was carried out for the rigid and baseline damped case, corresponding to a 1.9 N/mm spring stiffness. To reduce the required time, the other spring arrangements were only tested at 0°, considered as the most relevant direction. These include: 0.78 N/mm and 2.27 N/mm springs in the top two blocks, and a third configuration where the lower movable block was fixed, and the top module was allowed to move with a baseline spring stiffness of 1.9 N/mm. A close view of the damper and spring units in the model is shown in Fig. 4c. The test wind speeds at the reference height ranged from 5.5 m/s to 11 m/s. This corresponds to full-scale wind velocities of 22 m/s and 44 m/s, equivalent to the 1-year wind velocity at the reference height and 1.17 times the strength demand, respectively. Once the full-scale 10 min average wind speed reached the set-point, the response was recorded for 180 s (equivalent to 225 min at prototype scale) to ensure a robust response characterisation.

## Supplementary information

Supplementary Information

Description of Additional Supplementary Files

Supplementary Movie 1

Transparent Peer Review file

## Source data

Source Data

## Data Availability

Source data are provided with this paper. The raw wind tunnel test data generated in this study have been deposited in the Harvard Dataverse under the 10.7910/DVN/QDTAJL. Additional requests or queries can be directed to the corresponding author. Source data are provided with this paper.
