# Supplementary material for: Development of response-controlled tall buildings through own-mass mobilisation
Source: Nat Commun. 2026 Jun 24;17:7951. doi: 10.1038/s41467-026-74868-2 (PMC13448495; doi:10.1038/s41467-026-74868-2)
Supplement: Supplementary file 1 [file 41467_2026_74868_MOESM1_ESM.pdf]

SUPPLEMENTARY INFORMATION

Development of response-controlled tall buildings through own-mass mobilisation

Miguel Martinez-Paneda[1,2], Ahmed Y. Elghazouli[1,3,4*], Kevin Gouder[5] and William Algaard[2]

[1] Department of Civil & Environmental Engineering, Imperial College London, UK
[2] Arup, London, UK
[3] Department of Civil & Environmental Engineering, Hong Kong Polytechnic University, Hong Kong
[4] Nile University, Giza, Egypt
[5] Department of Aeronautics, Imperial College London, UK

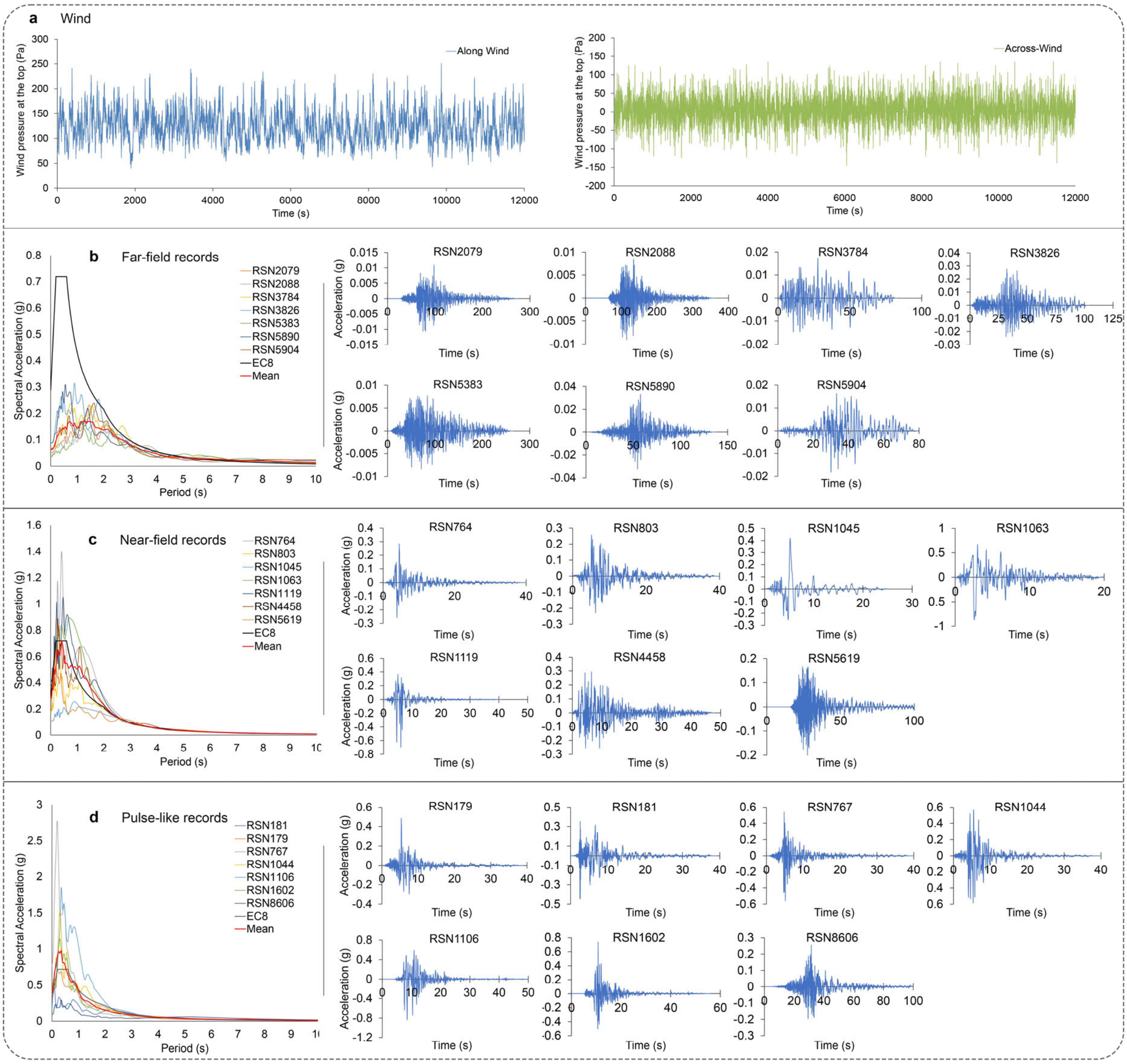

Supplementary Figure 1: Wind and seismic numerical inputs.

a, wind time history forcing function in the along and across-wind directions. **b,** scaled response spectrum and unscaled acceleration-time histories for the seven selected far-field earthquake records. **c,** scaled response spectrum and unscaled acceleration-time histories for the seven selected near-field records. **d,** scaled response spectrum and unscaled acceleration-time histories for the seven selected pulse-like records.

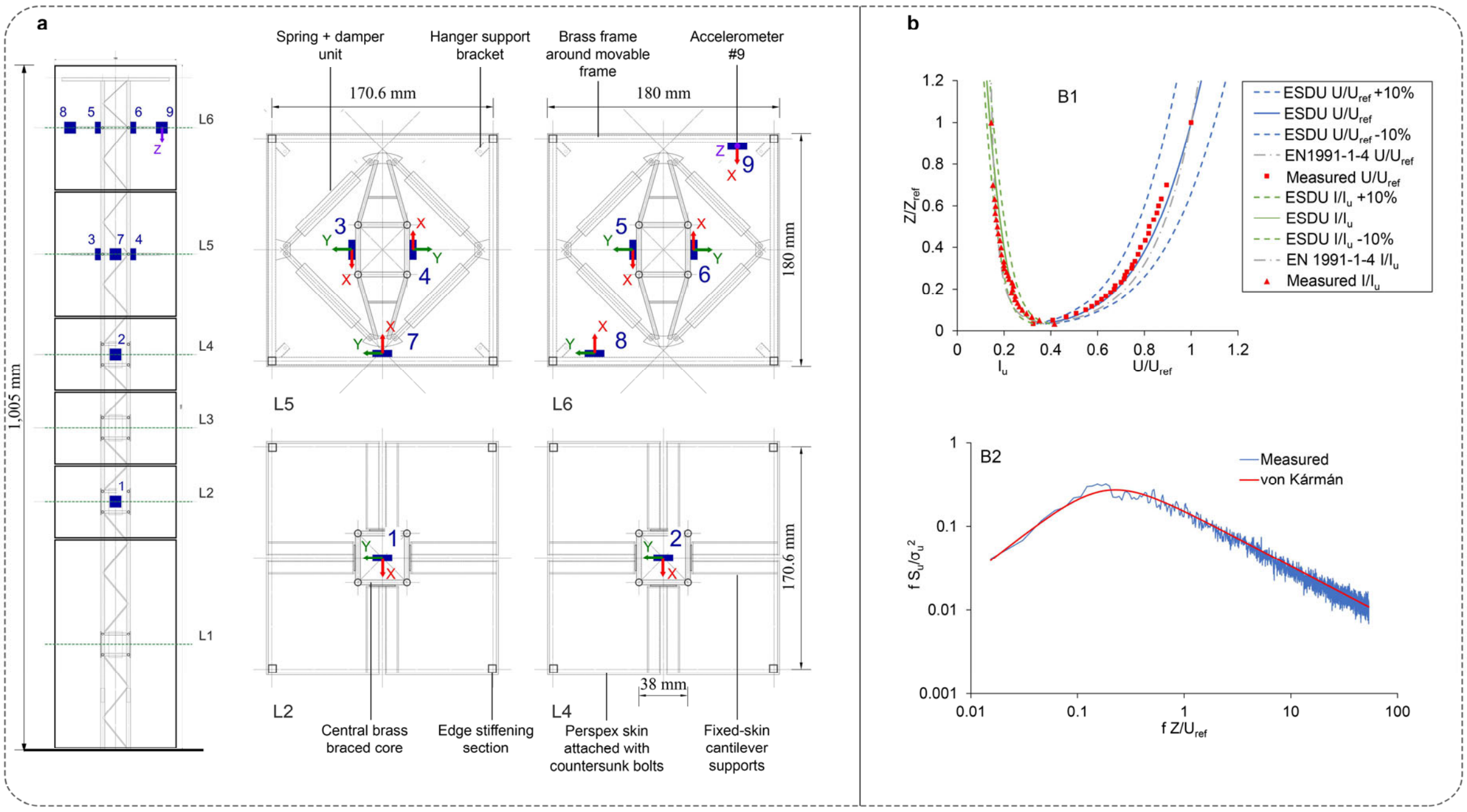

Supplementary Figure 2: Model instrumentation and wind simulation

a, accelerometer locations within the aeroelastic model, with their local axes. **b**, wind tunnel test simulated wind characteristics.

Supplementary Table 1: Dynamic properties of the prototype building and lumped mass FEM.

Mode	Prototype 3D FEM model				1D Prototype lumped mass FEM model			
	Period [s]	UX	UY	RZ	Period [s]	Difference	UX	Difference
1	9.9	36 %	23 %	0 %	10.3	4%	57%	3%
2	9.9	23 %	36 %	0 %	-	-	-	-
3	5.0	0 %	0 %	77 %	-	-	-	-
4	2.4	0 %	21 %	0 %	2.5	4%	20%	5%
5	2.4	21 %	0 %	0 %	-	-	-	-
6	1.9	0 %	0 %	12 %	-	-	-	-

Supplementary Table 2: Seismological data and scaling factor for the selected earthquake records.

Far-field records

NGA Record No.	Earthquake Name	Date	Magnitude	Distance to Fault (km)	V_{s30} (m/s)	PGA (g)	Scale Factor	MSE
RSN2079	Nenana Mountain, Alaska	2002	6.7	271.9	272	0.05	4.63	0.06
RSN2088	Nenana Mountain, Alaska	2002	6.7	272.9	342	0.04	4.47	0.06
RSN3784	Hector Mine	1999	7.1	251.5	351	0.07	3.97	0.07
RSN3826	Hector Mine	1999	7.1	211.7	308	0.09	3.16	0.04
RSN5383	Chuetshu-oki,	2007	6.8	215.2	230	0.04	4.23	0.12
RSN5890	El Mayor Cucapah, Mexico	2010	7.2	200.6	350	0.09	2.65	0.19
RSN5904	El Mayor Cucapah, Mexico	2010	7.2	227.5	340	0.07	3.61	0.20

Near-field records

NGA Record No.	Earthquake Name	Date	Magnitude	Distance to Fault (km)	V_{s30} (m/s)	PGA (g)	Scale Factor	MSE
RSN764	Loma Prieta	1989	6.9	11.1	271	0.41	1.53	0.01
RSN803	Loma Prieta	1989	6.9	9.3	348	0.18	0.68	0.02
RSN1045	Northridge-01	1994	6.7	5.5	286	0.11	0.26	0.01
RSN1063	Northridge-01	1994	6.7	6.5	282	0.37	0.42	0.01
RSN1119	Kobe, Japan	1995	6.9	0.3	312	0.41	0.59	0.03
RSN4458	Montenegro, Yugoslavia	1979	7.1	5.8	319	0.28	0.94	0.02
RSN5619	Iwate, Japan	2008	6.9	8.4	279	0.18	0.69	0.03

Pure pulse-like records

NGA Record No.	Earthquake Name	Date	Magnitude	Distance to Fault (km)	V_{s30} (m/s)	PGA (g)	Scale Factor	MSE
RSN179	Imperial Valley-06	1979	6.5	7.1	209	0.32	0.67	0.18
RSN181	Imperial Valley-06	1979	6.5	1.4	203	0.16	0.36	0.02
RSN767	Loma Prieta	1989	6.9	12.8	350	0.76	1.36	0.09
RSN1044	Northridge-01	1994	6.7	5.9	269	0.37	0.63	0.02
RSN1106	Kobe, Japan	1995	6.9	1.0	312	0.57	0.68	0.04
RSN1602	Duzce, Turkey	1999	7.1	12.0	294	0.39	0.53	0.03
RSN8606	El Mayor Cucapah, Mexico	2010	7.2	11.4	242	0.09	0.36	0.06

Supplementary Table 3: Simplified modal mass matching.

Module	Floors	Prototype height (m)	Model height (m)	ϕ	$\sum m_{prototype} \varphi^2$ (kg)	Scaled equiv. 1st mode model mass (kg)
6	54 to 65	279	0.93	0.879	17,202,430	0.823
5	42 to 63	225	0.75	0.617	8,793,323	0.856
4	35 to 41	175.5	0.585	0.407	2,178,850	0.485
3	28 to 34	144	0.48	0.289	1,127,699	0.497
2	21 to 27	112.5	0.375	0.188	496,019	0.516
1	GF to 20	49.5	0.165	0.047	217,990	4.2643
