# Supplementary material for: Development of response-controlled tall buildings through own-mass mobilisation
Source: Nat Commun. 2026 Jun 24;17:7951. doi: 10.1038/s41467-026-74868-2 (PMC13448495; doi:10.1038/s41467-026-74868-2)
Supplement: Supplementary file 2 [file 41467_2026_74868_MOESM2_ESM.pdf]

Description of Additional Supplementary Files

Supplementary Movie 1. Video illustrating typical wind tunnel test response for rigid and damped models
