# Supplementary material for: Development of response-controlled tall buildings through own-mass mobilisation
Source: Nat Commun. 2026 Jun 24;17:7951. doi: 10.1038/s41467-026-74868-2 (PMC13448495; doi:10.1038/s41467-026-74868-2)
Supplement: Supplementary file 3 [file 41467_2026_74868_MOESM4_ESM.pdf]

nature portfolio

Peer Review File

Development of response-controlled tall buildings through own-mass mobilisation

Corresponding Author: Professor Ahmed Elghazouli

Parts of this Peer Review File have been redacted as indicated to maintain the confidentiality of unpublished data.

This file contains all reviewer reports in order by version, followed by all author rebuttals in order by version.

Attachments originally included by the reviewers as part of their assessment can be found at the end of this file.

Version 0:

Reviewer comments:

Reviewer #1

(Remarks to the Author)
Please see the attached file.

Reviewer #2

(Remarks to the Author)
This manuscript describes a numerical and experimental study of a distributed tuned-mass damper (TMD) system in which some floors of a tall building are slightly decoupled from a stiffer structural core to act as discrete TMDs. These floors instead rest on an isolation device that presumably (see below) provides stiffness, and a set of viscous dampers that dissipates energy. The study uses a prototype 65-story, 300-m-tall building and a 1/300-scale model experimental specimen tested in a wind tunnel to demonstrate that the dynamic response -- in terms of floor accelerations and displacements in both wind and seismic response -- is reduced relative to that if the upper floors were rigidly connected to the core.

The manuscript is relevant, providing an interesting study that contributes to the body of knowledge of multiple TMD systems and, with some minor changes as listed below, is suitable for publication.

1. Reference 32 should be cited specifically; while Feng & Mita likely could not, more than 30 years ago, sufficiently simulate the complex behavior of a 65-story structure as in this manuscript, their idea to use parts of the structure on many floors poses the key concept of the current manuscript.

2. Further, studies of multiple tuned-mass dampers (MTMDs) should be cited as well, in two groups, those that examine multiple TMDs on a single primary structure (primarily to address multiple modes or the mistuning problem):
* Snowdon, Wolfe, & Kerlin, J. Acoust. Soc. Am., 1984
* Xu & Igusa, Earthq. Engrg. & Str. Dyn., 1992
* Igusa & Xu, J. Sound & Vibration, 1994
* Abe & Fujino, Earthq. Engrg. & Str. Dyn., 1994
* Kareem & Kline, J. Struct. Engrg., 1995

and those with multiple TMDs distributed across a structure
* Bergman, McFarland, Hall, Johnson, & Kareem, 5th Int'l. Conf. on Struct. Safety and Reliability, 1989
* Zapfe & Lesieutre, AIAA Journal, 1997
* Moon, Struct. Design Tall Spec. Build., 2010
* Fu & Johnson, J. Engrg. Mech., 2011
* Yang, Sedaghati, & Esmailzadeh, Struct. Control Health Monit., 2015

3. For the experimental apparatus, it is unclear why three different stiffness values are used. Presumably, the stiffnesses would be chosen so that the scale model matches some scaled versions of the full-scale model's first several natural

frequencies and mode shapes. The manuscript only reports the first mode shape.

4. There is no mention of the first torsional mode of the experimental specimen. It is often the first torsional mode that is particularly problematic (see, for example, the classic example of the John Hancock Tower, now called the 200 Clarendon St building, in Boston in the 1970s).

5. The PSD graphs in Fig. 5d would be easier to interpret if a second prototype frequency scale were added (i.e., keep the actual frequency scale, but add a second horizontal axis below it that shows the frequencies of the full-scale prototype corresponding to those of the scale model). Based on Extended Data Table 2, are the prototype frequencies presumably 1/75th of those of the scale model?

6. Isolation devices have been studied extensively for buildings, particularly as base isolation, but also for mid-story isolation [e.g., Kobayashi & Koh, J. Struct. and Constr. Engrg., AIJ, 2002]. However, one of the prime considerations is what happens if the interstory motion is larger than expected and one or more isolation devices fail. For the structure design envisioned in this paper, it seems that this would drop the entire weight of a story, now free from the central core that was previously supporting the gravity load, instead onto the story below; this, of course, could be catastrophic. While hard stoppers can be used to limit motion relative to the core, hitting them will induce significant vibratory shocks, typically with high-frequency content that would cause notable acceleration peaks. While the manuscript states that the excitations considered do not result in core-relative displacements sufficiently large to be problematic, some discussion would be warranted.

7. Fig. 2d shows the "sliding/elastomeric bearing" that provides the horizontal flexibility of an upper floor to move relative to the core. Sliding bearings and elastomeric bearings behave very differently, and elastomeric sliders (which incorporate both a rubber bearing above or below a sliding surface) behave yet differently still. Yet, there is no detail as to how these devices are modeled in the numerical assessment, nor is it clear how they are approximated in the scaled experimental specimen.

Reviewer #3

(Remarks to the Author)
This research questions the conventional assumption that tall buildings should be designed as rigid, static structures and instead introduces a framework that embraces and utilizes structural movement. To evaluate the proposed ideas, the study employed both numerical analysis and novel wind tunnel testing techniques. Nevertheless, substantial revisions are required to improve the clarity of the presented work and to enable future studies to effectively compare their findings with this research.
The reviewer suggests the following revisions to the manuscript:
1. In the abstract, it is not clear how this experiment is different from any TMD study? The abstract lacks info on the test novelty.
2. Still in the abstract, the authors abruptly introduce “seismic assessment”. With no clear explanation on what types of analysis were done.
3. Line 30-31: can the authors provide examples of locations or events where both wind and seismic loading are concurrently considered critical for tall buildings?
4. Lines 35-37: It would be good to refer to recent damage observations pertaining to wind effects on tall buildings and facades. A recent back-to-back extreme wind events were measured in Housten Texas. Some references to consider:
Eissa, M., Metwally, O., Alawode, K., Elawady, A., Lori, G., (2025) “Performance of high-rise building façades under wind loading: A state-of-the-art review”; Journal of Building Engineering; https://doi.org/10.1016/j.jobe.2025.114072
Kalliontzis, D., et al. (2024). StEER 2024 Houston Derecho Joint Preliminary Virtual Reconnaissance Report and Early Access Reconnaissance Report (PVRR-EARR). Retrieved from: https://www.designsafe-ci.org/data/browser/public/designsafe.storage.published/PRJ-4704/#detail-649770070724832785-242ac118-0001-012
Metwally, O., Ibrahim, H., Elawady, A., Zisis, I., Gan Chowdhury, A., (2025) “Wind load impact on tall building facades: damage observations during severe wind events and wind tunnel testing” Frontiers in Built Environment. Volume 10 – 2024,https://doi.org/10.3389/fbuil.2024.1514523.

5. In line 40-41, the authors refer to façade damage caused by seismic loading and the possibility for damage mitigation through damping systems. Can damping systems designed to mitigate seismic-induced vibrations also be used for wind-induced vibrations? This is also in line of my previous comments that the focus of this paper is not clear between seismic or wind loading and effects on tall buildings.
6. In the reference section, the references seem to be not listed properly. It starts at 53 rather than 1.
7. In lines 45-47: please clarify the reason for this conclusion regarding Tuned Mass Damper. “ mass systems are generally unable to reduce wind or seismic strength demands….”. if the damping system will function in away to reduce the excessive vibrations in a building, how it is not reducing the strength demand? Please also check these recent publications showing the efficiency of mass damping system:

Faisal Nissar Malik, Kolay Chinmoy. Optimal parameters for tall buildings with a single viscously damped outrigger considering earthquake and wind loads. Struct Des Tall Spec Build 2023;32(7). http://dx.doi.org/10.1002/tal.2003, e2003.

Malik, F., Ibrahim, H., Cao, L., Ricles, J., Elawady, A., Gan Chowdhury, A., (2026) “Real-time multi-directional aeroelastic hybrid simulation for tall building response under wind loading” Engineering Structures, V. 353, 122185; https://doi.org/10.1016/j.engstruct.2026.122185

8. In figure 1-f, the Japanese names together with the clarifications left there in English are not discussed in the text.

9. In lines 121-123: from functionality POV, how a moving floor can be adopted in everyday function of the building?
10. In lines 123-124: what is the justification of considering the wind load as a static load? Such simplification will not allow the proper examination of the proposed system. The simplification is also extended to the assumptions of the finite element model (FEM) which neglects the 3-D effects of the wind loading acting on A 3D tall building where both the along wind and across wind excitations need to be investigated. Maybe the authors can clarify that the numerical assessment was used before carrying out the aeroelastic wind tunnel test to help with the experimental program planning and design.
11. In line 113, the authors states that the third mode of vibration has no coupling with the first two. How was that decided. There is no proof of such a statement provided in the analysis.
12. In lines 136, the authors refer to along- and across-wind time histories use in the FEM. However, earlier, they said the load was static. Also, it is not clear how these time histories were generated. This section requires a thorough description of the assumptions used in the FEM.
13. Please revise the text describing Figure 2 to add more details, descriptions, to enhance the understanding of the proposed mechanism.
14. In line 151: what is meant by “Extended Data Fig. 1b”? The authors should clarify the data used in their analysis to enable future comparisons to their study.
15. In lines 158-159: the authors mention that seismic frequency content is larger, which is not expected, and thus yielded higher damper force: “The mean of the peak differential displacements remained low, at 83 mm, with a damper force of 668 kN, significantly larger than for wind, due to the higher frequency content.”. It would be useful to plot the power spectrum density of both events and highlight the frequency of the structure on the plot. This way, one can better understand why wind caused lesser damping forces.
16. How does the maximum drift compares to the code recommended values? Please provide a comment in the numerical analysis section.
17. Similar to the numerical assessment section, the wind tunnel testing lacks so many details, I will try to list them here:
- Which wind tunnel facility was used?
- What are the scaling ratios used to maintain similarity? At least the velocity scale is missing.
- What terrain exposure was used and what is the wind speed profile. How long was the test run for?
- What is the use of the lead mass on the first floor? Why the masses were not uniformly distributed along the height to represent the tall building of this study?
- What is the role of the FEM here, see line 182 “enabling full parametric definition of geometry”. According to figure 4, the model is skin is already defined which is partially fixed for the lower floors.
- It is important to provide validation plots for the aeroelastic model in the form of free vibration test results. How many modes were achieved? Figure 4.b shows the first mode shape achieved. However, missing validation information include frequencies and other modes (3rd mode?).

Version 1:

Reviewer comments:

Reviewer #1

(Remarks to the Author)
The authors properly addressed the comments.

Reviewer #2

(Remarks to the Author)
The authors edits and responses are acceptable for all of the first review's comments except for the last one, #7. There is still not even a mention in the text that there are sliding bearings, much less how the authors modeled them in the numerical analysis (e.g., was a perfect frictionless slider assumed? if so, how do the authors know that friction in the slider does not have a significant effect on performance? what parameters were used for the sliders?) nor how they are realized in the experimental model. Please add these details to the manuscript.

Reviewer #3

(Remarks to the Author)
I have no additional comments.

Open Access This Peer Review File is licensed under a Creative Commons Attribution 4.0 International License, which permits use, sharing, adaptation, distribution and reproduction in any medium or format, as long as you give appropriate credit to the original author(s) and the source, provide a link to the Creative Commons license, and indicate if changes were made.
In cases where reviewers are anonymous, credit should be given to 'Anonymous Referee' and the source.
The images or other third party material in this Peer Review File are included in the article’s Creative Commons license, unless indicated otherwise in a credit line to the material. If material is not included in the article’s Creative Commons license and your intended use is not permitted by statutory regulation or exceeds the permitted use, you will need to obtain permission directly from the copyright holder.
To view a copy of this license, visit https://creativecommons.org/licenses/by/4.0/

Authors' Response to Reviewers' Comments on NCOMMS-26-012048-T
"Development of response-controlled tall buildings through own-mass mobilisation"

Below is the authors' response to the reviewers' comments on the above manuscript. The reviewers' comments are shown in italics, whilst the authors' responses are shown in normal font style and preceded by "R". Changes are highlighted in yellow in the revised manuscript. We would like to thank the reviewers for their interest in the paper and their insightful and constructive suggestions which have appreciably improved the manuscript.

Reviewer #1

This paper proposes a novel concept for mitigating the vibration response of tall buildings by mobilizing the building's own mass as a damper mass, rather than installing additional external mass. Specifically, it suggests decoupling several upper floors from the central core to allow horizontal movement and connecting the core to these movable floors using springs and fluid viscous dampers (FVDs). This configuration aims to generate significant damping within a manageable range of relative displacement.

The proposed technique was applied to a 65-story (300 m) example building through numerical analysis to evaluate wind and seismic responses. Furthermore, to verify the effectiveness of the concept, experiments were conducted using a 1:300 scale multi-degree-of-freedom (MDOF) aeroelastic wind tunnel model. The results from both numerical analysis and wind tunnel tests demonstrate that not only accelerations (serviceability index) but also base shear and overturning moments (strength indices) can be significantly reduced. Simultaneously, the relative displacement between the core and the movable floors is shown to be manageable within a range acceptable for MEP (mechanical, electrical, and plumbing) and finishing details.

While response mitigation in tall buildings is typically discussed with a focus on target acceleration requirements, this study presents a refreshing perspective by mobilizing the building's own mass for damping, effectively utilizing the building's motion as a design asset. The direct validation through aeroelastic wind tunnel experiments, in addition to numerical analysis, is particularly impressive.

However, the following points must be addressed to improve the clarity and completeness of the manuscript.

R: The authors are gratified by the positive comments of the reviewer and appreciate the constructive and helpful comments which are addressed in detail below.

1. In the "Introduction" and the section on "Large own-mass damping", it is stated that mass systems such as TMDs generally cannot reduce the strength demand of wind and earthquake loads. However, if a mass system effectively reduces acceleration and dynamic responses, it could potentially reduce inertia forces and the base shear, leading to a reduction in strength demand. The authors should clarify whether this statement implies that TMDs with typical mass ratios (approx. 1~2%) struggle to significantly reduce strength-level demands, or if there is another intent regarding code requirements or reliability perspectives.

R: As pointed out by the reviewer, TMDs could theoretically reduce strength demands under first mode excitation. However, as a structure approaches its ultimate strength demands, it would experience an increase in flexibility due to additional cracking in core walls and coupling beams, and steel yielding. This increase in flexibility would shift the first natural period, thus leading to a reduced performance of the TMD, with even a potential negative influence, due to detuning effects. As single devices, TMDs also lack sufficient redundancy to

cover maintenance or locked-in periods that the building would rely on for ultimate strength demands. Lines 65 to 69 of the revised manuscript have been amended to clarify thus, as follows: "However, despite their benefits[18,31], TMDs have several limitations including their inability to control seismic demands due to frequency tuning as well as their shortcomings in reducing strength demands due to their lack of redundancy, as the system could be under maintenance in addition to possible detuning risks at the vibration levels associated with ultimate strength demands."

2. This study sets the upper 1/3 of the building as the movable portion. What is the rationale behind selecting this specific ratio? It would be beneficial to know if a parameter study was conducted regarding performance, relative displacement, and constructability trade-offs for other ratios (e.g., 1/4 or 1/2), or if this choice was based on practical design constraints, such as the location of specific transition or plant floors.

R: As the reviewer notes, selecting one-third of the height was to align with the MEP plant floor, which, with flexible cladding systems and higher detailing allowances, can readily accommodate the larger inter-storey drifts associated with the transition floor. For clarification, the following text has been added to line 131 of the manuscript: "Part of the own mass of the building was mobilised as a damper mass by allowing the floors to move from the core along the top third by providing a horizontal movement joint at floor level (Fig. 2b and Fig. 2d). While the study also investigates the option of mobilising the top sixth of the building, the presence of the plant floor at two thirds of the height creates a natural position for accommodating the larger inter-storey drifts associated with the transition floor."

3. In Figure 3, a local peak in maximum drift is observed near the transition floor. While this is likely due to the change in boundary conditions from the fixed section to the movable section, there is a concern regarding whether this local deformation could cause repetitive loading or fatigue in structural elements (connections, joints, diaphragms, etc.) or affect overall stability through increased P-delta effects in actual applications.

R: The reviewer raises an important issue that needs clarification. As highlighted in Figs 3.a and 3.b, the drifts at the MEP transition floor are generally within h/100, above what curtain wall facades can typically accommodate (h/250 to h/400), but well within the design allowance for a specific detail that can be easily incorporated in MEP floors, due to their requirement to have louvres. When crossing over movement joints or for large drifts, these can incorporate sliding head or bottom details, telescopic mullions or compressible gaskets, which can generally accommodate drift demands of 1% to 1.5% with current detailing and well below the PEER TBI and the 2023 LATBSDC guidelines of 0.03 limit. Although the small relative displacement would not lead to additional P-Delta effects when compared to those already present in the building and for which the horizontal springs could be designed for. In a similar manner, given the low differential displacements expected under service conditions, in the order of h/200, the gravity columns are not expected to be subjected to significant fatigue demands. While larger demands would take place under moderate and extreme seismic events, these would not differ from conventional demands on a moment frame under seismic events. This would either require the columns to be designed with adequate ductility to avoid local buckling and subsequent rupture, or with a physical pin, a sliding joint, or a triple-friction pendulum base at the transition floor.

A more concise clarification of the above issue is incorporated at Line 144 of the revised manuscript, as follows: "Implementation is achieved by treating the interface between the mobilised floors and the core as a conventional movement joint sized to the design relative displacement demand. At floor level, standard movement joint assemblies such as cover plates and fire-rated compressible infills, preserve compartmentation while permitting the required

motion. Gravity loads are transferred to the core by connecting the floor framing to perimeter corbels with sliding joints, designed to accommodate the maximum expected movements (Fig. 2d). The helicoidal springs around the damper units ensure a robust lifecycle and provide lateral stability at both service and ultimate conditions. MEP systems crossing the interface are designed using typical flexible couplings and expansion loops according to conventional high-rise detailing practices, typically employed to accommodate thermal movements, and without the need to incorporate any bespoke components."

Further clarification is also added at Line 186, as follows: "The different behaviour of the lower, fixed floors and the movable portion is evident at the transition floor. The peak inter-storey drift at this level remains below the maximum inter-storey drift observed at the top of the building in all conditions. It also remains below the ultimate drift limit of 0.03[51], which can be readily accommodated within the louvred façade of the MEP floor using conventional detailing practices."

4. In the Experimental methodology section, the wind speed range at the top of the model is given as 22 m/s~50 m/s, with 50 m/s described as 33% higher than the ultimate strength demand. Conversely, the Wind field simulation under Methods presents a full-scale wind speed range of 22 m/s~44 m/s, stating that 44 m/s is 1.33 times the strength demand. These two descriptions appear to be contradictory. A consistency check and correction regarding the wind speed range and the 1.33 times the strength demand statement are required.

R: We thank the reviewer for spotting this. The strength wind demand at the reference height (300 m at prototype scale, or 1 m at the model scale), corresponds to a wind speed of 37.4 m/s. Consequently, 44 m/s is only 1.17 times the strength wind speed. This has now been corrected in the Methods section as follows: "This corresponds to full-scale wind velocities of 22 m/s and 44 m/s, equivalent to the 1-year wind velocity at the reference height and 1.17 times the strength demand, respectively."

5. The aeroelastic wind tunnel experiments were conducted using three candidate spring stiffnesses. The rationale for selecting these specific values is not sufficiently explained in the text. Furthermore, the results suggest that the vibration reduction effect is greatest at the lowest stiffness. It would enhance the practical applicability of the research if the authors could clarify whether performance consistently increases as stiffness decreases (or if an optimum point exists) and provide guidelines for selecting stiffness during the design stage.

R: The reviewer raises an important point that needs clarification. The third paragraph of the Experimental Methodology section (from Line 241) has been revised as follows: "The spring–damper assemblies were interchangeable to allow three different representative stiffness values (0.78 N/mm, 1.9 N/mm and 2.27 N/mm at model scale). These correspond to horizontal stiffnesses and differential displacement under the EN 1991-1-4 static wind load per floor at the prototype scale of 882 kN/m and 410 mm, 2,150 kN/m and 170 mm, and 2,568 kN/m and 140 mm, respectively) and the locking of one or both movable modules, enabling direct comparison with a rigid counterpart. The optimal spring stiffness for the system would correspond to that where the frequency of the mobilised mass is tuned to match the frequency of the building, as a conventional TMD. However, this is not generally feasible due to its low value, as it would result in unacceptable relative movements. Accordingly, every specific application requires calibration of the lower spring stiffness and corresponding maximum relative displacements that can be realistically incorporated."

6. Although the proposed idea is innovative and achieves high vibration reduction without significantly increasing additional mass, implementing movable joints, flexible MEP connections, and fire/disaster-rated joints for the upper floors may significantly increase construction complexity and costs compared to traditional techniques like TMDs. To support

the claim that this system can be implemented within a practical workflow, the authors' perspective on potential construction/maintenance risks and economic feasibility should be added.

R: This requires clarification as pointed out by the reviewer. As noted in the response to Point 3 above, this has now been incorporated from Line 144 as follows: "Implementation is achieved by treating the interface between the mobilised floors and the core as a conventional movement joint sized to the design relative displacement demand. At floor level, standard movement joint assemblies such as cover plates and fire-rated compressible infills, preserve compartmentation while permitting the required motion. Gravity loads are transferred to the core by connecting the floor framing to perimeter corbels with sliding joints, designed to accommodate the maximum expected movements (Fig. 2d). The helicoidal springs around the damper units ensure a robust lifecycle and provide lateral stability at both service and ultimate conditions. MEP systems crossing the interface are designed using typical flexible couplings and expansion loops according to conventional high-rise detailing practices, typically employed to accommodate thermal movements, and without the need to incorporate any bespoke components."

7. While the concept is novel, it could also be interpreted as a form of internal partial base isolation, as it dynamically decouples a portion of the upper mass from the core. To help establish the academic positioning of this work, the authors should more clearly distinguish the fundamental differences between this technique and traditional base isolation from their own perspective.

R: As noted by the reviewer, in a traditional base isolation system, the building mass is decoupled from the base excitation, hence there are some broadly general similarity. However, there are notable differences which are now highlighted in the manuscript in Line 180, as follows: "As the damped floors are dynamically decoupled from the base excitation, the movable tier showed more pronounced improvements, with inter-storey drifts reducing by 52% and accelerations by up to 74% when averaged over the decoupled floors. While this behaviour somewhat generally resembles that of base isolated buildings, albeit these are ineffective in tall buildings due to their higher loads and longer first natural period, the response control mechanism also has a reciprocal beneficial effect in the remaining structure, reducing overall demands." In addition, the following is included from Line 206: "The results highlight the potential of the system in enhancing tall building safety, efficiency and resilience, significantly reducing non-structural damage and downtime under both frequent and large wind and seismic events. Furthermore, the results also illustrate the combined energy dissipation mechanism of the system. For long-period content, such as wind or far-field records, around the first building vibration period, the decoupled mass mitigates overall response by behaving akin to a detuned mass damper, with a slight phase offset, reducing generalised forces with a minimal stroke. For short excitation periods, such as for near-field or pulse-like records, which engage the higher vibration modes, energy is dissipated through the differential motions at both ends of the dampers, resembling the behaviour of a distributed damping system. This dual action, enabled by the large mobilised mass, explains the observed broadband effectiveness and tolerance to detuning across both wind and seismic excitations."

8. In Lines 76–78, the authors state that "The benefits of mobilising a large damper mass have been explored in several seismic-oriented TMD variations, although these are hindered by constructability and integration constraints including large relative displacement and tuning requirements." However, the present study proposes mobilizing a portion of the building's own mass as the damper mass. In this regard, the manuscript would benefit from a more detailed and engineering-oriented discussion on the practical feasibility of such a concept in real structural design. In particular, issues related to constructability, structural

integration, and implementation within realistic design constraints should be addressed more explicitly. For example, the brief description of the spring stiffness setting provided in Line 123 is insufficient for readers to clearly assess whether the proposed configuration can be realistically implemented. Additional clarification on how this system could be designed, detailed, and realized in practice would significantly improve the credibility and applicability of the proposed approach.

R: We would like to thank the reviewer for emphasising this important aspect. In combination with previous comments, this was incorporated in the manuscript by adding an additional paragraph at the end of the Practical Implementation section in Line 144 as follows:

"Implementation is achieved by treating the interface between the mobilised floors and the core as a conventional movement joint sized to the design relative displacement demand. At floor level, standard movement joint assemblies such as cover plates and fire-rated compressible infills, preserve compartmentation while permitting the required motion. Gravity loads are transferred to the core by connecting the floor framing to perimeter corbels with sliding joints, designed to accommodate the maximum expected movements (Fig. 2d). The helicoidal springs around the damper units ensure a robust lifecycle and provide lateral stability at both service and ultimate conditions. MEP systems crossing the interface are designed using typical flexible couplings and expansion loops according to conventional high-rise detailing practices, typically employed to accommodate thermal movements, and without the need to incorporate any bespoke components." Furthermore, the reference spring stiffness provided in Line 123 is further clarified with the following addition (now at Line 135): "The spring stiffness was set to allow a maximum differential displacement of 150 mm under the 50-year Eurocode 1 wind load applied as a static load[49], as a representative first value for preliminary design purposes."

9. In the Practical Implementation section, the authors state that an MDOF model was analyzed using SAP2000. However, the description of the structural model and analysis procedure is currently insufficient for technical evaluation. The manuscript only reports the fundamental period of the building, while essential information regarding the structural system, modeling assumptions, and analysis settings is missing. In particular, it remains unclear whether linear or nonlinear time-history analyses were performed, and no details are provided regarding modeling parameters, boundary conditions, damping assumptions, or solver configurations. Without such information, readers cannot assess the validity or reproducibility of the numerical implementation.

R: We thank the reviewer for raising this. The key characteristics have now been also been summarised within the Methods section, from Line 501, as follows:

"The 300 m central core building was modelled within the finite element software SAP2000[50] using a MDOF representation where the mass of very floor at the core and the office floor were represented as a single degree of freedom. The simplified lumped mass model was benchmarked to the detailed 3D model of the building, replicating the total mass and mass distribution, and achieving a close match between the first three translational modal periods[47]. The dynamic properties of the prototype building and lumped mass model are shown in Extended Data Table 1. Within the model, the damper elements were represented following the Maxwell model of viscoelasticity, with a linear spring in series with a nonlinear dashpot to capture the effect of the damper and brace stiffness in the formulation, and a relaxation time constant of 0.01. Due to the non-classical damping arrangement of the system and FVDs velocity-dependency, the system response was investigated using nonlinear time-history analysis with direct integration."

10. Furthermore, in the Numerical Assessment section, the authors claim to employ "realistic wind scenarios" and "realistic seismic scenarios." At minimum, for the wind loading

case, the manuscript should clearly present the adopted wind velocity profile and turbulence characteristics. Providing figures or explicit descriptions of the inflow wind profile, turbulence model, or spectral representation would be necessary to substantiate the claim of realism. Simply stating that numerical analyses were conducted under realistic conditions, without documenting the loading assumptions, results in insufficient technical justification.

R: This requires clarification as noted by the reviewer. The wind simulations adopted correspond to realistic time histories generated for the design of a recently completed tall building in Taipei. While full details cannot be included, additional explanation has been added in the Methods section, Line 516, with a reference to this in the main section of the text, to clarify, as follows: "The 300 m central core building was subjected to a pair of wind tunnel time-history forcing functions consisting of an along-wind and an across-wind time history record for a 10-minute mean wind speed of 24.5 m/s at 10 m, and an hourly mean of 40.8 m/s at 600 m. This corresponds to a wind event for a 50-year return period with hurricane conditions with an upwind suburban terrain around the 360 degrees of the building, and a wind climate model based on local surface wind measurements at Taipei Songshan Airport, with the corresponding wind profile for Taipei as stipulated in the Taiwan Building Code 2007 (Extended Data Fig. 1a)." We would also wish to emphasise that although specific wind conditions are relevant to a particular location, the purpose of the numerical assessment was to show the behavioural trends by comparing the response of a rigid and a damped counterparts.

11. If the above information cannot be adequately provided, it may be more appropriate for the authors to position the study primarily around the aeroelastic wind tunnel testing described from the Experimental Methodology section onward, where the originality and completeness of the work appear to be better supported by clearly documented experimental procedures.

R: We thank the reviewer for raising this. While we agree that the aeroelastic wind tunnel is instrumental to the work and offers the best representation, the parallelism of the results with the numerical simulations adds another layer of comparison benefits of the overall system and can assist readers to perform initial numerical investigations for preliminary assessments.

12. Finally, in the description of Figure 1, the Japanese temple Horyu-ji is misspelled as Hoyru-ji. Considering the academic nature of this paper, the spelling in the figure caption should be corrected.

R: We thank the reviewer for spotting this typo. This has now been corrected in the manuscript.

Reviewer #2

This manuscript describes a numerical and experimental study of a distributed tuned-mass damper (TMD) system in which some floors of a tall building are slightly decoupled from a stiffer structural core to act as discrete TMDs. These floors instead rest on an isolation device that presumably (see below) provides stiffness, and a set of viscous dampers that dissipates energy. The study uses a prototype 65-story, 300-m-tall building and a 1/300-scale model experimental specimen tested in a wind tunnel to demonstrate that the dynamic response -- in terms of floor accelerations and displacements in both wind and seismic response -- is reduced relative to that if the upper floors were rigidly connected to the core.

The manuscript is relevant, providing an interesting study that contributes to the body of knowledge of multiple TMD systems and, with some minor changes as listed below, is suitable for publication.

R: The authors are gratified by the positive comments of the reviewer and appreciate the constructive and helpful comments which are addressed in detail below. The authors would also like to emphasise that additional information has now been added in the revised manuscript to clarify how the proposed system differs from Tuned Mass Dampers or multiple TMDs.

1. Reference 32 should be cited specifically; while Feng & Mita likely could not, more than 30 years ago, sufficiently simulate the complex behavior of a 65-story structure as in this manuscript, their idea to use parts of the structure on many floors poses the key concept of the current manuscript.

R: As noted by the reviewer, while the energy dissipating mechanism differs from that proposed herein, the work by Feng & Mitta is relevant and worth of further mention. This has now been incorporated from Line 84, as follows: "Feng and Mitta proposed the "mega subconfiguration" concept, aimed at creating usable interior substructures within the building and either have them vibrate in shear with diagonal fluid viscous dampers (FVDs) or tuned to vibrate as a conventional TMD[35]."

2. Further, studies of multiple tuned-mass dampers (MTMDs) should be cited as well, in two groups, those that examine multiple TMDs on a single primary structure (primarily to address multiple modes or the mistuning problem):

** Snowdon, Wolfe, & Kerlin, J. Acoust. Soc. Am., 1984*
** Xu & Igusa, Earthq. Engrg. & Str. Dyn., 1992*
** Igusa & Xu, J. Sound & Vibration, 1994*
** Abe & Fujino, Earthq. Engrg. & Str. Dyn., 1994*
** Kareem & Kline, J. Struct. Engrg., 1995*

and those with multiple TMDs distributed across a structure

** Bergman, McFarland, Hall, Johnson, & Kareem, 5th Int'l. Conf. on Struct. Safety and Reliability, 1989*
** Zapfe & Lesieutre, AIAA Journal, 1997*
** Moon, Struct. Design Tall Spec. Build., 2010*
** Fu & Johnson, J. Engrg. Mech., 2011*
** Yang, Sedaghati, & Esmailzadeh, Struct. Control Health Monit., 2015*

R: The references suggested by the reviewer have now been added to the revised manuscript as References 36 to 45 and cited accordingly into the text.

3. For the experimental apparatus, it is unclear why three different stiffness values are used. Presumably, the stiffnesses would be chosen so that the scale model matches some scaled versions of the full-scale model's first several natural frequencies and mode shapes. The manuscript only reports the first mode shape.

R: We would like to clarify that, while an optimum performance would be achieved if the spring stiffness were to match the optimal frequency, like a conventional TMD, this would lead to excessive relative displacements for practical implementation and would thus prevent a usable portion of the building from being mobilised as a damper mass. Rather than proposing another variation of a conventional TMD/MTMD, the proposed concept sets the stiffness of the spring not to the optimum to maximise equivalent damping, but to control serviceability displacements. Accordingly, the three differential stiffnesses are selected as representative stiffnesses that would return relative displacements between the core and the office floors within the range that could be implemented with conventional detailing.

This is explained in Lines 106 to 117 as follows: "Considerable levels of damping could potentially be generated by allowing a significant portion of the building own mass to move relative to the lateral stability system, while being connected together through springs and fluid viscous dampers (FVDs). The FVDs ensure that accelerations within the movable portion remain within comfort levels[13], thus enabling a usable part of the building to be mobilised as a damper mass. The spring stiffness is not tuned to match optimal tuning as an orthodox TMD, but to resist the static component of the wind load and control differential displacements within serviceability conditions. Rather than adding extra mass, as in a conventional TMD, the proposed concept capitalises on the large inherent mass present in tall buildings and the large displacements they can experience at the top under service conditions to convert them into a design asset. This allows floors in tall buildings to experience minimal differential displacements with respect to the lateral stability system to provide substantially enhanced dynamic performance."

As suggested by the reviewer, additional explanations have now also been included in the third paragraph at the Experimental Methodology section, Line 241, to provide further clarity, has follows: "The spring–damper assemblies were interchangeable to allow three different representative stiffness values (0.78 N/mm, 1.9 N/mm and 2.27 N/mm at model scale). These correspond to horizontal stiffnesses and differential displacement under the EN 1991-1-4 static wind load per floor at the prototype scale of 882 kN/m and 410 mm, 2,150 kN/m and 170 mm, and 2,568 kN/m and 140 mm, respectively) and the locking of one or both movable modules, enabling direct comparison with a rigid counterpart. The optimal spring stiffness for the system would correspond to that where the frequency of the mobilised mass is tuned to match the frequency of the building, as a conventional TMD. However, this is not generally feasible due to its low value, as it would result in unacceptable relative movements. Accordingly, every specific application requires calibration of the lower spring stiffness and corresponding maximum relative displacements that can be realistically incorporated."

4. There is no mention of the first torsional mode of the experimental specimen. It is often the first torsional mode that is particularly problematic (see, for example, the classic example of the John Hancock Tower, now called the 200 Clarendon St building, in Boston in the 1970s).

R: While torsion can play a significant role in certain cases, this was not determined to be the case for the current application. As the reviewer points out, the John Hancock Tower has a rectangular plan, thus leading to its torsional response. This is not applicable to the current design due to the lack of coupling between the torsional and translational modes as noted in the "Practical Implementation" section, with the first translational modes having a 9.9 s period, and the third, torsional mode, with a 5 s period. Following the reviewer's suggestion, the dynamic properties of the prototype 3D FEM are now included in the Extended Data Table 1.

The comparison between the torsional model of the prototype and that of the model is also shown in Extended Data Table 5. Even though it would be ideal to accurately match the torsional mode to that of the prototype, due to the requirement to have the mass of the top office floors at the skin, rather than uniformly distributed, the aeroelastic model has a higher rotational inertia, leading to a slight decrease in the torsional stiffness. This is noted in Lines 595 and 669. Thanks to the large separation between the translational and torsional modes in both the prototype and the aeroelastic model, and the square shape of the building, the torsional response has a nearly-negligible effect on the response. It may also be worth noting that, irrespective of this difference, the wind tests showed that the damping system resulted in a significant reduction in torsional demands.

5. The PSD graphs in Fig. 5d would be easier to interpret if a second prototype frequency scale were added (i.e., keep the actual frequency scale, but add a second horizontal axis below it that shows the frequencies of the full-scale prototype corresponding to those of the scale

model). Based on Extended Data Table 2, are the prototype frequencies presumably 1/75th of those of the scale model?

R: As noted by the reviewer, the prototype frequencies are 1/75th of those of the scaled model as per the table. On the other hand, it is worth noting that Figure 5 is currently aligned to the time histories on the left and in matching size to the ones below. After testing Figure 5 with a second axis, the visibility of the graph and its alignment with other plots will reduce, which would be detrimental to its appearance and clarity.

6. Isolation devices have been studied extensively for buildings, particularly as base isolation, but also for mid-story isolation [e.g., Kobayashi & Koh, J. Struct. and Constr. Engrg., AIJ, 2002]. However, one of the prime considerations is what happens if the interstory motion is larger than expected and one or more isolation devices fail. For the structure design envisioned in this paper, it seems that this would drop the entire weight of a story, now free from the central core that was previously supporting the gravity load, instead onto the story below; this, of course, could be catastrophic. While hard stoppers can be used to limit motion relative to the core, hitting them will induce significant vibratory shocks, typically with high-frequency content that would cause notable acceleration peaks. While the manuscript states that the excitations considered do not result in core-relative displacements sufficiently large to be problematic, some discussion would be warranted.

R: As suggested by the reviewer, additional information has now been included in the revised manuscript to clarify this. While the system is designed to remain fully operational under wind, low and moderate seismic events, the joint should be designed to accommodate MCE_R amplitude levels without pounding or risk of catastrophic collapse. MEP services and finishes crossing the interface are accepted to experience damage, yet maximum relative displacements should be controlled to ensure no uncontrolled pounding between both masses. While a larger gap from the MCE_R expected displacement can be provided, the FVD stroke length or shear keys would ultimately ensure overall vertical stability for cases beyond MCE_R, and thus guarantee no-collapse beyond what would be required for a typical high-rise building. To clarify this further, the following text has been added to Line 192: "Although the level of relative displacements largely depends on the site seismicity, for areas of high seismicity, it is expected that the system would be subjected to larger differential displacements under moderate and extreme events. Accordingly, while it is common practice to accommodate damage in finishes and MEP services at those levels, the movement joint gap should be designed to accommodate the displacements expected from the Risk-Targeted Maximum Considered Earthquake (MCE_R) without pounding, and to avoid disproportionate collapse in all instances."

7. Fig. 2d shows the "sliding/elastomeric bearing" that provides the horizontal flexibility of an upper floor to move relative to the core. Sliding bearings and elastomeric bearings behave very differently, and elastomeric sliders (which incorporate both a rubber bearing above or below a sliding surface) behave yet differently still. Yet, there is no detail as to how these devices are modeled in the numerical assessment, nor is it clear how they are approximated in the scaled experimental specimen.

R: We thank the reviewer for pointing this out. The connection was denotated as sliding/elastomeric bearings to show the versatility of the system, with the potential of the sliding bearing (as described in the text), being replaced in an alternative application by an elastomeric bearing that could work together with the FVD to provide damping and horizontal stiffness. As the reviewer correctly points out, this can be confusing to a reader, hence the figure has been revised to show only a sliding bearing (ETFE+SS), which is the one considered in the study. Figure 2d (Detail D2) has been updated accordingly in the revised manuscript.

Reviewer #3

This research questions the conventional assumption that tall buildings should be designed as rigid, static structures and instead introduces a framework that embraces and utilizes structural movement. To evaluate the proposed ideas, the study employed both numerical analysis and novel wind tunnel testing techniques. Nevertheless, substantial revisions are required to improve the clarity of the presented work and to enable future studies to effectively compare their findings with this research.

R: The authors are grateful by the positive comments of the reviewer and appreciate the constructive and helpful comments which are addressed in detail below.

The reviewer suggests the following revisions to the manuscript:

1. In the abstract, it is not clear how this experiment is different from any TMD study? The abstract lacks info on the test novelty.

R: We thank the reviewer for pointing this out. To address this, we have modified the first sentence of the abstract as follows: "This paper describes a novel structural response reduction approach that, rather than adding mass as in conventional mass damping techniques, takes advantage of the large inherent mass present within a building and uses its own mass to generate large levels of reliable damping with minimal differential movements and without the need for frequency tuning". We would also like to note that the journal imposes a strict limit on the length of the abstract, hence we have highlighted this briefly. It is then complemented by more detailed explanations in the subsequent section of the paper as discussed in the responses below.

2. Still in the abstract, the authors abruptly introduce "seismic assessment". With no clear explanation on what types of analysis were done.

R: We thank the reviewer for pointing this out. To address this, we have modified the penultimate sentence of the abstract as follows: "The results of the wind tunnel tests, coupled with complementary numerical studies as well seismic time-history simulations, demonstrate the considerable promise of the proposed system for drastically reducing the demands on the superstructure and foundation. We would also like to note that the journal imposes a strict limit on the length of the abstract, hence we have highlighted this briefly. It is then complemented by more detailed explanations in the subsequent section of the paper as discussed in the responses below.

3. Line 30-31: can the authors provide examples of locations or events where both wind and seismic loading are concurrently considered critical for tall buildings?

R: We thank the reviewer for raising this. Wind loads tend to govern building stiffness requirements due to the requirement to control accelerations, inter-storey drifts and overall building deformations within the service level. This is especially the case for buildings within the range of 200 m to 600 m. Seismic demands at MCE_R govern structural detailing, ductility requirements and total base shear. This however, changes drastically depending on location. The authors have experience of designing tall buildings in parts of the world where both are critical: Philippines and Taipei (with typhoons and high-seismicity), Shenzhen (with typhoons and low seismicity although still governing at MCE_R-level), Mexico City (with low winds and high seismicity, with wind still governing service conditions), or Tokyo (with typhoons and very high seismicity). Further information is available from: W. Algaard et al., 'Investigating Interactions Between Wind and Seismic Actions for Tall Buildings', SECED Vol. 28 (2) 2017.

4. Lines 35-37: It would be good to refer to recent damage observations pertaining to wind effects on tall buildings and facades. A recent back-to-back extreme wind events were measured in Housten Texas. Some references to consider:

Eissa, M., Metwally, O., Alawode, K., Elawady, A., Lori, G., (2025) "Performance of high-rise building façades under wind loading: A state-of-the-art review"; Journal of Building Engineering; https://doi.org/10.1016/j.jobe.2025.114072
Kalliontzis, D., et al. (2024). StEER 2024 Houston Derecho Joint Preliminary Virtual Reconnaissance Report and Early Access Reconnaissance Report (PVRR-EARR). Retrieved from: https://www.designsafe-ci.org/data/browser/public/designsafe.storage.published/PRJ-4704/#detail-649770070724832785-242ac118-0001-012
Metwally, O., Ibrahim, H., Elawady, A., Zisis, I., Gan Chowdhury, A., (2025) "Wind load impact on tall building facades: damage observations during severe wind events and wind tunnel testing" Frontiers in Built Environment. Volume 10 – 2024,https://doi.org/10.3389/fbuil.2024.1514523.

R: We thank the reviewer for pointing out these three suggested relevant references reporting damage from wind events. These have now been added as References 23 to 25 and cited in Line 43 of the revised manuscript.

5. In line 40-41, the authors refer to façade damage caused by seismic loading and the possibility for damage mitigation through damping systems. Can damping systems designed to mitigate seismic-induced vibrations also be used for wind-induced vibrations? This is also in line of my previous comments that the focus of this paper is not clear between seismic or wind loading and effects on tall buildings.

R: As raised by the reviewer, redundant damping systems can generally mitigate both wind and seismic demands depending on their calibration. TMDs cannot reduce seismic or wind strength demands due to their specific frequency tunning to the first mode, susceptibility to detuning due to changes in dynamic properties associated with building damage during a seismic event and lack of redundancy. However, distributed systems such as Fluid Viscous Dampers (Torre Mayor in Mexico, Costums Residential Tower in Auckland, 181 Fremont Street in SF, or the St Francis Shangri-Pa Place in Manila) or viscoelastic coupling beams (454 Yonge in Toronto) among many others, can mitigate both wind and seismic demands as distributed systems. Buckling Restrained Braces or Friction devices are generally be set out to remain elastic under wind and only dissipate energy once the design wind load is exceeded.

The response control mechanism proposed in this paper is able to reduce wind and service strength demands and as such is examined herein under both loading conditions. The main focus is on the wind response as the most critical and that generally governs preliminary sizing, but the benefits extend to both under service and ultimate strength conditions. This explains the double focus in the manuscript.

6. In the reference section, the references seem to be not listed properly. It starts at 53 rather than 1.

R: The references are listed properly, but this misunderstanding arises from the journal style requirements. The reference section for the main section of the revised manuscript starts at Line 349 (previously at Line 291). The numbering then continues with additional references after the Methods section, starting at Line 745 in the revised manuscript (previously at Line 633). The split between both corresponds to the Nature portfolio journals style requirements.

7. In lines 45-47: please clarify the reason for this conclusion regarding Tuned Mass Damper. "mass systems are generally unable to reduce wind or seismic strength demands….". if the damping system will function in away to reduce the excessive vibrations in a building,

how it is not reducing the strength demand? Please also check these recent publications showing the efficiency of mass damping system:

Faisal Nissar Malik, Kolay Chinmoy. Optimal parameters for tall buildings with a single viscously damped outrigger considering earthquake and wind loads. Struct Des Tall Spec Build 2023;32(7). http://dx.doi.org/10.1002/tal.2003*, e2003.*

Malik, F., Ibrahim, H., Cao, L., Ricles, J., Elawady, A., Gan Chowdhury, A., (2026) "Real-time multi-directional aeroelastic hybrid simulation for tall building response under wind loading" Engineering Structures, V. 353, 122185; https://doi.org/10.1016/j.engstruct.2026.122185

R: As pointed out by the reviewer, TMDs could theoretically reduce strength demands under first mode excitation. However, as a structure approaches its ultimate strength demands, it would experience an increase in flexibility due to additional cracking in core walls and coupling beams, and steel yielding. This increase in flexibility would shift the first natural period, thus leading to a reduced performance of the TMD, with even a potential negative influence, due to detuning effects. As single devices, TMDs also lack sufficient redundancy to cover maintenance or locked-in periods that the building would rely on for ultimate strength demands. Lines 65 to 69 of the revised manuscript have been amended to clarify thus, as follows: "However, despite their benefits[18,31], TMDs have several limitations including their inability to control seismic demands due to frequency tuning as well as their shortcomings in reducing strength demands due to their lack of redundancy, as the system could be under maintenance in addition to possible detuning risks at the vibration levels associated with ultimate strength demands."

8. In figure 1-f, the Japanese names together with the clarifications left there in English are not discussed in the text.

R: As pointed out by the reviewer, this has been incorporated by updating the reference to Fig. 1f in Lines 99 to 104 as follows: "In light of the above discussion, and with the aim of developing a novel large-mass response-reduction approach, inspiration was initially drawn from the architecture of traditional Japanese temples, in which different components, within the *Kumimono,* dissipate seismic energy by experiencing small differential motions and rotations relative to each other, only restrained by the *Shinbashira* (or central timber pillar), which has a further energy dissipation mechanism sliding and lifting over the *Shinso* (or heart stone) as shown in Fig. 1f[46]."

9. In lines 121-123: from functionality POV, how a moving floor can be adopted in everyday function of the building?

R: As noted in the manuscript, given the low accelerations present in the movable floors (below human perception) and low motion levels, users would be insensitive to the movements. Following the reviewer's comment, the following text has been added to the end of the "Practical Implementation" section from Line 144 to clarify in a concise manner how the system is incorporated into a building: "Implementation is achieved by treating the interface between the mobilised floors and the core as a conventional movement joint sized to the design relative displacement demand. At floor level, standard movement joint assemblies such as cover plates and fire-rated compressible infills, preserve compartmentation while permitting the required motion. Gravity loads are transferred to the core by connecting the floor framing to perimeter corbels with sliding joints, designed to accommodate the maximum expected movements (Fig. 2d). The helicoidal springs around the damper units ensure a robust lifecycle and provide lateral stability at both service and ultimate conditions. MEP systems crossing the

interface are designed using typical flexible couplings and expansion loops according to conventional high-rise detailing practices, typically employed to accommodate thermal movements, and without the need to incorporate any bespoke components."

10. In lines 123-124: what is the justification of considering the wind load as a static load? Such simplification will not allow the proper examination of the proposed system. The simplification is also extended to the assumptions of the finite element model (FEM) which neglects the 3-D effects of the wind loading acting on A 3D tall building where both the along wind and across wind excitations need to be investigated. Maybe the authors can clarify that the numerical assessment was used before carrying out the aeroelastic wind tunnel test to help with the experimental program planning and design.

R: To clarify, as noted in the "Numerical assessment" and "Aeroelastic wind tunnel results" sections, the wind loads are considered as dynamic loads. Indication of the "code static wind load" is only used for reference purposes due to its ease of reference for preliminary design of the spring elements. To avoid confusion, this has now been clarified in the text by extending the sentence between Lines 135 and 138 as follows: "The spring stiffness was set to allow a maximum differential displacement of 150 mm under the 50-year Eurocode 1 wind applied as a static load[49], as a representative first value for preliminary design purposes."

As suggested by the reviewer, clarification of the purpose of the numerical wind assessments is now incorporated in the first paragraph of the "Numerical assessment" section (from Line 156), as follows: "The numerical simulations described in this section provide more detailed insights into the dynamic response under realistic wind and seismic scenarios using the MDOF lumped-mass model (Fig. 3) (with further information given in the Methods section). These studies also provide additional insights to steer subsequent experimental assessments and further illustrate the benefits of decoupling a usable portion of the building and mobilising its own mass as a damper mass."

11. In line 113, the authors states that the third mode of vibration has no coupling with the first two. How was that decided. There is no proof of such a statement provided in the analysis.

R: The design of the prototype building was calibrated to ensure such dynamic behaviour. As noted by the reviewer, for clarification, an additional table has now been included within the Methods section as "Extended Data Table 1: Dynamic properties of the prototype building and lumped mass FEM." This shows no coupling between the third mode and the first two. The table is also cited to in Line 509 of the revised manuscript, as follows: The dynamic properties of the prototype building and lumped mass model are shown in Extended Data Table 1."Further information on the design of the prototype building can be found in Ref 47 as noted in line 117: "The proposed approach was considered within an illustrative 65-floor, 300 m tall composite building, featuring a steel-braced central core (Fig. 2)[47],"

12. In lines 136, the authors refer to along- and across-wind time histories use in the FEM. However, earlier, they said the load was static. Also, it is not clear how these time histories were generated. This section requires a thorough description of the assumptions used in the FEM.

R: As noted in the response to Point 10 above, the code static wind load was only employed for preliminary design of the spring stiffness for ease of reference. This is particularly important as designers do not typically have access to wind tunnel results or time histories for a specific building, hence the static code wind load can provide a reference point for preliminary estimates of the potential of this decoupled arrangement in their buildings. To avoid confusion, Line 135 onwards has been modified as follows: "The spring stiffness was set to allow a maximum differential displacement of 150 mm under the 50-year Eurocode 1 wind load applied as a static load[49], as a representative first value for preliminary design purposes."

The wind simulations adopted correspond to realistic time histories generated for the design of a recently completed tall building in Taipei. While full details cannot be included, additional explanation has been added in the Methods section, with a reference to this in the main section of the text, for clarification, as follows: "The numerical simulations described in this section provide more detailed insights into the dynamic response under realistic wind and seismic scenarios using the MDOF lumped-mass model (Fig. 3) (with further information available in the Methods section)." And, in the Methods section (Line 516), as follows: "The 300 m central core building was subjected to a pair of wind tunnel time-history forcing functions consisting of an along-wind and an across-wind time history record for a 10-minute mean wind speed of 24.5 m/s at 10 m, and an hourly mean of 40.8 m/s at 600 m. This corresponds to a wind event for a 50-year return period with hurricane conditions with an upwind suburban terrain around the 360 degrees of the building, and a wind climate model based on local surface wind measurements at Taipei Songshan Airport, with the corresponding wind profile for Taipei as stipulated in the Taiwan Building Code 2007 (Extended Data Fig. 1a). To adapt to the different building dimensions and speed, the wind force was proportionally increased to match the slightly increased prototype building width and peak amplitude to match a top displacement of H/500 as the design target. The decoupled arrangement was investigated using a static stiffness defined to enable a maximum differential displacement of up to 150 mm under the EN 1991-1-4 50-year static wind load36 and with an optimal damper coefficient to maximise first mode damping for the selected stiffness. The response was evaluated by comparing a damped or decoupled arrangement with a conventionally rigid counterpart in terms of five performance indices, namely: top displacement, floor-by-floor accelerations, inter-storey drifts, damper forces and differential displacements, as the key indices describing tall building response and directly related to the required structural sizes."

13. Please revise the text describing Figure 2 to add more details, descriptions, to enhance the understanding of the proposed mechanism.

R: We thank the reviewer for pointing this out. Additional explanations have now been included (Line 131 onwards) as follows: "Part of the own mass of the building was mobilised as a damper mass by allowing the floors to move from the core along the top third by providing a horizontal movement joint at floor level (Fig. 2b and Fig. 2d). While the study also investigates the option of mobilising the top sixth of the building, the presence of the plant floor at two thirds of the height creates a natural position for accommodating the larger inter-storey drifts associated with the transition floor. The spring stiffness was set to allow a maximum differential displacement of 150 mm under the 50-year Eurocode 1 wind load applied as a static load[49], as a representative first value for preliminary design purposes. The building response was then examined in SAP2000[50] using a multi-degree-of-freedom (MDOF) lumped mass model incorporating the mass of the core and the individual floors with their respective stiffnesses (Fig. 2c). Using the half-power bandwidth method, the equivalent modal damping was found to increase from the intrinsic value of 0.8% to about 6% with the proposed arrangement. This level far exceeds the 0.5%-2% typically attainable with conventional TMDs and common damping arrangements[18,31].

"Implementation is achieved by treating the interface between the mobilised floors and the core as a conventional movement joint sized to the design relative displacement demand. At floor level, standard movement joint assemblies such as cover plates and fire-rated compressible infills, preserve compartmentation while permitting the required motion. Gravity loads are transferred to the core by connecting the floor framing to perimeter corbels with sliding joints, designed to accommodate the maximum expected movements (Fig. 2d). The helicoidal springs around the damper units ensure a robust lifecycle and provide lateral stability at both service and ultimate conditions. MEP systems crossing the interface are designed using typical flexible

couplings and expansion loops according to conventional high-rise detailing practices, typically employed to accommodate thermal movements, and without the need to incorporate any bespoke components."

14. In line 151: what is meant by "Extended Data Fig. 1b"? The authors should clarify the data used in their analysis to enable future comparisons to their study.

R: We would like to clarify that, according to the journal requirements, the manuscript has a "Methods" section appended to it where additional explanatory information, figures and tables are included. The terms "Extended Data Figure" and "Extended Data Table" are required by the Nature Communication style guidelines.

15. In lines 158-159: the authors mention that seismic frequency content is larger, which is not expected, and thus yielded higher damper force: "The mean of the peak differential displacements remained low, at 83 mm, with a damper force of 668 kN, significantly larger than for wind, due to the higher frequency content.". It would be useful to plot the power spectrum density of both events and highlight the frequency of the structure on the plot. This way, one can better understand why wind caused lesser damping forces.

R: We thank the reviewer for highlighting the need for further clarification. Typically, seismic records have higher frequency ranges compared to wind events. This can for example be visualised in the Boggs & Dragovich response spectra shown in Fig. R1 below.

[REDACTED]

Fig. R1 Typical Boggs & Dragovich response spectra

While this is typical, as suggested by the reviewer, there is a need to provide clarification in the manuscript of its influence on the response. Given that seismic records have a higher energy content in the higher frequencies and that wind has a lower energy content in the low frequencies, it is important to explain how the system reacts to this difference. The PSD for the WTT can be found on Fig.5d, and the response spectra for the seismic records in Extended Data Fig. 1. In addition, the following explanation has now been added in the manuscript, from Line 206 onwards, as follows: "The results highlight the potential of the system in enhancing tall building safety, efficiency and resilience, significantly reducing non-structural damage and downtime under both frequent and large wind and seismic events. Furthermore, the results also illustrate the combined energy dissipation mechanism of the system. For long-period content, such as wind or far-field records, around the first building vibration period, the decoupled mass mitigates overall response by behaving akin to a detuned mass damper, with a slight phase offset, reducing generalised forces with a minimal stroke. For short excitation periods, such as for near-field or pulse-like records, which engage the higher vibration modes, energy is dissipated through the differential motions at both ends of the dampers, resembling the behaviour of a distributed damping system. This dual action, enabled by the large mobilised

mass, explains the observed broadband effectiveness and tolerance to detuning across both wind and seismic excitations."

16. How does the maximum drift compares to the code recommended values? Please provide a comment in the numerical analysis section.

R: We thank the reviewer for raising this important point, as the transition floor does experience large drifts. While these inter-storey drifts are lower than those exhibited at the top of a rigid counterpart under the same excitation, as shown in Fig. 3b, it is still worth discussing it relative to the PEER TBI drift limit. Additional information has therefore been added in Line 186 as follows: "The different behaviour of the lower, fixed floors and the movable portion is evident at the transition floor. The peak inter-storey drift at this level remains below the maximum inter-storey drift observed at the top of the building in all conditions. It also remains below the ultimate drift limit of 0.03[51], which can be readily accommodated within the louvred façade of the MEP floor using conventional detailing practices."

17. Similar to the numerical assessment section, the wind tunnel testing lacks so many details, I will try to list them here:
- Which wind tunnel facility was used?
- What are the scaling ratios used to maintain similarity? At least the velocity scale is missing.
- What terrain exposure was used and what is the wind speed profile. How long was the test run for?
- What is the use of the lead mass on the first floor? Why the masses were not uniformly distributed along the height to represent the tall building of this study?
- What is the role of the FEM here, see line 182 "enabling full parametric definition of geometry". According to figure 4, the model is skin is already defined which is partially fixed for the lower floors.
- It is important to provide validation plots for the aeroelastic model in the form of free vibration test results. How many modes were achieved? Figure 4.b shows the first mode shape achieved. However, missing validation information include frequencies and other modes (3rd mode?).

R: As noted in the response to Point 14 above, we would like to clarify that, according to the journal requirements and guidelines, the manuscript has a "Methods" section appended to it where additional explanatory information, figures and tables are included.

We of course fully agree with the reviewer that all this information is essential. We would like to confirm that it is all included in the "Methods" section at the end of the paper (after the initial reference set), as required by the Nature Communication style guidelines. The listed details information are specifically provided in the "Methods" section within the "Aeroelastic model formulation and design", "Model fabrication, instrumentation and validation" and "Wind field simulation" chapters and associated figures and tables.

To avoid any confusion to the readers, an additional statement has been included in the first part of the manuscript (Line 279) to make sure that reference is made to the Methods section, as follows: "Additional information on the formulation, fabrication, and validation of the aeroelastic model and wind field simulation can be found in the Methods section."

Finally, we would like to thank the three reviewers again for their insightful and helpful comments, which have been addressed within the revised manuscript, and which we believe have appreciably improved the quality and clarity of the paper.

Authors' Response to Reviewers' Comments on NCOMMS-26-012048A
"Development of response-controlled tall buildings through own-mass mobilisation"

Below is the authors' response to the reviewers' comments on the above manuscript. The reviewers' comments are shown in italics, whilst the authors' responses are shown in normal font style and preceded by "R". Changes are highlighted in yellow in the revised manuscript. We would like to thank the reviewers again for their interest in the paper and their insightful and constructive suggestions.

Reviewer #1

The authors properly addressed the comments.

R: The authors thank the reviewer for the endorsement of the revised manuscript.

Reviewer #2

The authors edits and responses are acceptable for all of the first review's comments except for the last one, #7. There is still not even a mention in the text that there are sliding bearings, much less how the authors modeled them in the numerical analysis (e.g., was a perfect frictionless slider assumed? if so, how do the authors know that friction in the slider does not have a significant effect on performance? what parameters were used for the sliders?) nor how they are realized in the experimental model. Please add these details to the manuscript.

R: We thank the reviewer for emphasising this point, which requires further discussion. We have clarified the gravity-support assumptions in both the main text and the Methods sections. In the numerical model, gravity loads are carried by perimeter columns idealised as nominally pinned (gravity-only) members, with the floor masses coupled via rigid links/diaphragm constraints or springs and dampers working in parallel (as per Figure 2c and Reference 47). No explicit sliding-bearing element (and hence no friction parameter) was introduced in the SAP2000 model, as the study focuses on the global dynamic response governed by the spring-damper links. In the aeroelastic model, the mobilised modules are suspended from a propped cantilever to provide vertical support while avoiding parasitic friction and unintended lateral coupling at 1:300 scale. The manuscript now states this explicitly as per the following additional extracts: In Line 140: "Office floor gravity loads are supported by the perimeter columns idealised as nominally pinned members, with the floor masses linked through rigid diaphragm constraints, consistent with the prototype modelling assumptions (Fig. 2c)[47]." In Line 512: "Vertical gravity loads at the office floors are transferred through the perimeter columns, which are idealised as nominally pinned (gravity-only) members. The floor masses are coupled via rigid links and springs and dampers, as appropriate, to represent the effect of the system while avoiding unintended participation of the gravity system in the lateral stiffness, consistently with the design of the prototype building." In Line 522: "Additional information on the numerical idealisation can be found elsewhere[47]." In Line 619: "The final aeroelastic model consists of a brass central core, four fixed facade modules that cantilever from the central core, and two top movable modules that hang from a propped cantilever that extends from the core, as illustrated in Fig. 4. Suspending the movable modules provides the required vertical support while allowing the spring–damper units to govern the horizontal coupling. This avoids parasitic friction and inadvertent outrigger effects that would be difficult to control at 1:300 scale and could otherwise alter the intended modal properties and energy dissipation mechanism."

Reviewer #3

I have no additional comments.

R: The authors thank the reviewer for the endorsement of the revised manuscript.

Title: Development of response-controlled tall buildings through own-mass mobilisation

This paper proposes a novel concept for mitigating the vibration response of tall buildings by mobilizing the building's own mass as a damper mass, rather than installing additional external mass. Specifically, it suggests decoupling several upper floors from the central core to allow horizontal movement and connecting the core to these movable floors using springs and fluid viscous dampers (FVDs). This configuration aims to generate significant damping within a manageable range of relative displacement.

The proposed technique was applied to a 65-story (300 m) example building through numerical analysis to evaluate wind and seismic responses. Furthermore, to verify the effectiveness of the concept, experiments were conducted using a 1:300 scale multi-degree-of-freedom (MDOF) aeroelastic wind tunnel model. The results from both numerical analysis and wind tunnel tests demonstrate that not only accelerations (serviceability index) but also base shear and overturning moments (strength indices) can be significantly reduced. Simultaneously, the relative displacement between the core and the movable floors is shown to be manageable within a range acceptable for MEP (mechanical, electrical, and plumbing) and finishing details.

While response mitigation in tall buildings is typically discussed with a focus on target acceleration requirements, this study presents a refreshing perspective by mobilizing the building's own mass for damping, effectively utilizing the building's motion as a design asset. The direct validation through aeroelastic wind tunnel experiments, in addition to numerical analysis, is particularly impressive.

However, the following points must be addressed to improve the clarity and completeness of the manuscript.

1. In the “Introduction” and the section on “Large own-mass damping”, it is stated that mass systems such as TMDs generally cannot reduce the strength demand of wind and earthquake loads. However, if a mass system effectively reduces acceleration and dynamic responses, it could potentially reduce inertia forces and the base shear, leading to a reduction in strength demand. The authors should clarify whether this statement implies that TMDs with typical mass ratios (approx. 1~2%) struggle to significantly reduce strength-level demands, or if there is another intent regarding code requirements or reliability perspectives.
2. This study sets the upper 1/3 of the building as the movable portion. What is the rationale behind selecting this specific ratio? It would be beneficial to know if a parameter study was conducted regarding performance, relative displacement, and constructability trade-offs for other ratios (e.g., 1/4 or 1/2), or if this choice was based on practical design constraints, such as the location of specific transition or plant floors.
3. In Figure 3, a local peak in maximum drift is observed near the transition floor. While this is likely due to the change in boundary conditions from the fixed section to the movable section, there is a concern regarding whether this local deformation could cause repetitive loading or fatigue in structural elements (connections, joints, diaphragms, etc.) or affect overall stability through increased P-delta effects in actual applications.
4. In the Experimental methodology section, the wind speed range at the top of the model is given as 22 m/s~50 m/s, with 50 m/s described as 33% higher than the ultimate strength demand. Conversely, the Wind field simulation under Methods presents a full-scale wind speed range of 22 m/s~44 m/s, stating that 44 m/s is 1.33 times the strength demand. These two descriptions appear

to be contradictory. A consistency check and correction regarding the wind speed range and the 1.33 times the strength demand statement are required.

5. The aeroelastic wind tunnel experiments were conducted using three candidate spring stiffnesses. The rationale for selecting these specific values is not sufficiently explained in the text. Furthermore, the results suggest that the vibration reduction effect is greatest at the lowest stiffness. It would enhance the practical applicability of the research if the authors could clarify whether performance consistently increases as stiffness decreases (or if an optimum point exists) and provide guidelines for selecting stiffness during the design stage.
6. Although the proposed idea is innovative and achieves high vibration reduction without significantly increasing additional mass, implementing movable joints, flexible MEP connections, and fire/disaster-rated joints for the upper floors may significantly increase construction complexity and costs compared to traditional techniques like TMDs. To support the claim that this system can be implemented within a practical workflow, the authors' perspective on potential construction/maintenance risks and economic feasibility should be added.
7. While the concept is novel, it could also be interpreted as a form of internal partial base isolation, as it dynamically decouples a portion of the upper mass from the core. To help establish the academic positioning of this work, the authors should more clearly distinguish the fundamental differences between this technique and traditional base isolation from their own perspective.
8. In Lines 76–78, the authors state that "*The benefits of mobilising a large damper mass have been explored in several seismic-oriented TMD variations, although these are hindered by constructability and integration constraints including large relative displacement and tuning requirements.*" However, the present study proposes mobilizing a portion of the building's own mass as the damper mass. In this regard, the manuscript would benefit from a more detailed and engineering-oriented discussion on the practical feasibility of such a concept in real structural design. In particular, issues related to constructability, structural integration, and implementation within realistic design constraints should be addressed more explicitly. For example, the brief description of the spring stiffness setting provided in Line 123 is insufficient for readers to clearly assess whether the proposed configuration can be realistically implemented. Additional clarification on how this system could be designed, detailed, and realized in practice would significantly improve the credibility and applicability of the proposed approach.
9. In the Practical Implementation section, the authors state that an MDOF model was analyzed using SAP2000. However, the description of the structural model and analysis procedure is currently insufficient for technical evaluation. The manuscript only reports the fundamental period of the building, while essential information regarding the structural system, modeling assumptions, and analysis settings is missing. In particular, it remains unclear whether linear or nonlinear time-history analyses were performed, and no details are provided regarding modeling parameters, boundary conditions, damping assumptions, or solver configurations. Without such information, readers cannot assess the validity or reproducibility of the numerical implementation.
10. Furthermore, in the Numerical Assessment section, the authors claim to employ "realistic wind scenarios" and "realistic seismic scenarios." At minimum, for the wind loading case, the manuscript should clearly present the adopted wind velocity profile and turbulence characteristics. Providing figures or explicit descriptions of the inflow wind profile, turbulence model, or spectral representation would be necessary to substantiate the claim of realism. Simply stating that numerical analyses were conducted under realistic conditions, without documenting the loading assumptions, results in insufficient technical justification.
11. If the above information cannot be adequately provided, it may be more appropriate for the authors to position the study primarily around the aeroelastic wind tunnel testing described from the

Experimental Methodology section onward, where the originality and completeness of the work appear to be better supported by clearly documented experimental procedures.

12. Finally, in the description of Figure 1, the Japanese temple Horyu-ji is misspelled as Hoyru-ji. Considering the academic nature of this paper, the spelling in the figure caption should be corrected.
